# Variable admittance control with sEMG-based support for wearable wrist exoskeleton

**DOI:** 10.3389/fnbot.2025.1562675

**Published:** 2025-09-01

**Authors:** Charles Lambelet, Melvin Mathis, Marc Siegenthaler, Jeremia P. O. Held, Daniel Woolley, Olivier Lambercy, Roger Gassert, Nicole Wenderoth

**Affiliations:** ^1^Neural Control of Movement Lab, Department of Health Sciences and Technology, Institute of Human Movement Sciences and Sport, ETH Zurich, Zürich, Switzerland; ^2^CRPP Stroke, Department of Neurology, University of Zurich, Zürich, Switzerland; ^3^Rehabilitation Engineering Laboratory, Department of Health Sciences and Technology, Institute of Robotics and Intelligent Systems, ETH Zurich, Zürich, Switzerland

**Keywords:** variable admittance control, surface electromyography, gravity compensation, stroke rehabilitation, wrist exoskeleton, wearables, visuomotor task, proprioceptive feedback

## Abstract

**Introduction:**

Wrist function impairment is common after stroke and heavily impacts the execution of daily tasks. Robotic therapy, and more specifically wearable exoskeletons, have the potential to boost training dose in context-relevant scenarios, promote voluntary effort through motor intent detection, and mitigate the effect of gravity. Portable exoskeletons are often non-backdrivable and it is challenging to make their control safe, reactive and stable. Admittance control is often used in this case, however, this type of control can become unstable when the supported biological joint stiffens. Variable admittance control adapts its parameters dynamically to allow free motion and stabilize the human-robot interaction.

**Methods:**

In this study, we implemented a variable admittance control scheme on a one degree of freedom wearable wrist exoskeleton. The damping parameter of the admittance scheme is adjusted in real-time to cope with instabilities and varying wrist stiffness. In addition to the admittance control scheme, sEMG- and gravity-based controllers were implemented, characterized and optimized on ten healthy participants and tested on six stroke survivors.

**Results:**

The results show that (1) the variable admittance control scheme could stabilize the interaction but at the cost of a decrease in transparency, and (2) when coupled with the variable admittance controller the sEMG-based control enhanced wrist functionality of stroke survivors in the most extreme angular positions.

**Discussion:**

Our variable admittance control scheme with sEMG- and gravity-based support was most beneficial for patients with higher levels of impairment by improving range of motion and promoting voluntary effort. Future work could combine both controllers to customize and fine tune the stability of the support to a wider range of impairment levels and types.

## 1 Introduction

Upper limb paresis is a common impairment poststroke affecting 75% of stroke survivors (Rathore et al., 2002). This manifests not only during whole arm movements but also during tasks that require well-coordinated hand and wrist actions. Wrist function in particular is essential in many activities of daily living (ADL) for orientating and stabilizing the hand (Palmer et al., 1985), and recovery of this function can have a meaningful impact on the quality of life poststroke (Squeri et al., 2013). Robot-mediated rehabilitation has the potential to provide intensive, repetitive, and task-specific training in a motivating environment (Norouzi-Gheidari et al., 2012; Colombo et al., 2007). Moreover, by supporting movements with the impaired limb, robotic training promotes voluntary effort and enhances proprioceptive feedback, which stimulates neuroplasticity in the neural circuitry that generates skilled movements (Miall et al., 2018; Ghez et al., 1990; Hasan, 1992). One limitation of most robotic rehabilitation devices to date is that they are stationary and require supervision from trained professionals. This could be overcome by portable exoskeletons that support the impaired limb based on the movement intention of the user (Lenzi et al., 2012), or by reducing the effect of gravity (Wu et al., 2016; Moubarak et al., 2010). The putative advantage of portable exoskeletons is that they would allow the integration of rehabilitation training into functional everyday tasks, which would provide a high training dose via distributed sessions in task-relevant contexts (Brewer et al., 2007; Bützer et al., 2019; Gasser et al., 2017).

Currently, there are only a few active wearable devices targeting wrist function that could be used as portable rehabilitation tools (Dragusanu et al., 2020; Choi et al., 2019; Higuma et al., 2017; Sangha et al., 2016; Al-Fahaam et al., 2016; Bartlett et al., 2015; Andrikopoulos et al., 2015; Khokhar et al., 2010), and none of these solutions are fully portable and mobile. Existing wearable wrist exoskeletons fall into two main categories. The first includes designs that use highly flexible and compliant structures, however, the control of these devices is challenging so they usually only provide relatively basic support, e.g., via pre-programmed (Dragusanu et al., 2020; Bartlett et al., 2015; Andrikopoulos et al., 2015) or remotely triggered (Higuma et al., 2017) movement sequences. The second category includes designs that use rigid structures which are less comfortable to wear but allow the implementation of more precise and continuous control schemes that incorporate physiological signals such as force myography (FMG) (Sangha et al., 2016) or surface electromyography (sEMG) (Khokhar et al., 2010). Control schemes based on physiological signals afford some level of voluntary effort from the stroke survivor, which is believed to be more beneficial for neural reorganization and recovery (Lotze et al., 2003; Perez et al., 2004). Moreover, precise and proportional control of the mechanical support enhances the rehabilitation of coordinated movements (Lotze et al., 2003; Ghez et al., 1990). These assumptions motivated the development of new interactive robot-based treatments that require active participation (Song et al., 2013; Hu et al., 2015; Lenzi et al., 2012).

One challenge when designing wearable actuated exoskeletons for poststroke rehabilitation is the development of an appropriate controller. sEMG is a technique that has been used extensively for the control of upper-limb robotic devices by decoding the user's movement intention (Farina et al., 2014). This method is non-invasive, easy to apply, and contains rich information about motor intentions. Moreover, the occurrence of the sEMG signal starts about 20–50 ms before overt movement takes place (Norman and Komi, 1979), which might help reduce delays in the robot's actuation system. However, the signal varies substantially across subjects and electrode placement, which requires a calibration after the device has been donned (Fleischer and Hommel, 2007; Hashemi et al., 2014; Lin et al., 2024). Typically, the signal is heavily filtered to extract its envelope, which is suitable for robotic control (Lyu et al., 2020). From there, the amplitude of the envelope is extracted to proportionally control joint velocity (Corbett et al., 2011), pressure (Sawicki and Ferris, 2009) or torque of the assistive device. Torque mapping is by far the most common and straightforward approach to obtain natural motion (Song et al., 2013; Hu et al., 2015; Lenzi et al., 2012), but precise control can be challenging due to the non-linear sEMG-torque relationship with respect to movement velocity and joint position (Farina, 2006; Solomonow et al., 1991).

An alternative control strategy that does not attempt to decode the user's movement intention is gravity compensation. By definition, a wearable exoskeleton moves with its user, and thus the mechanical support it provides to the limb is altered by the effect of gravity. One solution that takes this effect into account is evaluating the spatial orientation of the exoskeleton and adjusting the mechanical support accordingly. This can be done by implementing an inertial measurement unit (IMU) on the device. Gravity compensation has mostly been implemented on stationary exoskeletons that support shoulder and elbow joints to compensate for the weight of the arm (Hsieh et al., 2015; Spagnuolo et al., 2015). Anti-gravity support benefits upper-limb rehabilitation primarily by reducing the amount of fatigue experienced by acute and sub-acute patients, thus enabling an increase in the quality and dose of training (Kwakkel and Meskers, 2013). It is also effective for improving motor control and decreasing spasticity (Prange et al., 2006; Brewer et al., 2007).

Wearable exoskeletons featuring a rigid structural design with a direct implementation of the actuation system (i.e., DC motors) usually lack backdrivability. This results from the implementation of small actuators with high gear ratios in order to minimize weight. In this context, a natural and smooth physical human-robot interaction (pHRI) cannot solely be mediated through physiological signals or gravity support. The pHRI must be safe, but also reactive and compliant to the user's movement intentions (De Santis et al., 2008). To that end, a straightforward approach is to measure interaction forces with sensors connected in series with the kinematics chain (Nef et al., 2006). Admittance control is an appropriate and commonly adopted force-based control to actuate the robot (Kilic, 2017; Topini et al., 2022; Zeng and Hemami, 1997) as opposed to impedance control which is used with position-based and usually backdrivable systems. However, admittance control is prone to instability, especially when the human joint becomes stiffer during pHRI (Landi et al., 2017; Wang et al., 2015). For instance, oscillations of the end-effector can arise during a reaching task when the wrist needs to stiffen to stabilize the hand for a grasp. A possible strategy to prevent oscillations and instability is to dampen the system by adjusting the parameters of the admittance control scheme. A simplistic approach is to set these parameters sufficiently high in order to constantly dampen the pHRI, however, the transparency of the robotic system, i.e., its capacity to not apply resistance or assistance to free motion (Proietti et al., 2016), is then affected. The capability of haptic rehabilitation devices to provide transparent behavior is important for quantitatively assessing the patient's ability to perform movements without being disturbed by the device dynamics (Tagliamonte et al., 2011). Thus, in order to allow free motion, but at the same time stabilize the pHRI, the admittance control parameters need to be adapted dynamically. A variable admittance control scheme adapts its parameters either by detecting the instability (Landi et al., 2017; Dimeas and Aspragathos, 2016) or by estimating the stiffness of the human limb (Castellini et al., 2014; Raiano et al., 2020).

The present work investigates a novel approach combining variable admittance control for sEMG-based and gravity-based support implemented on a wearable and non-backdrivable wrist exoskeleton. In this context, we test whether (1) variable admittance control will stabilize the pHRI while allowing transparent motion, and (2) the sEMG-based and gravity-based controllers will enhance wrist functionality and promote voluntary effort. For this purpose, we performed a proof of concept study and implemented different control strategies using a 1 DOF device actively supporting wrist extension and flexion movements (Lambelet et al., 2020, 2017). The controllers were assessed in a visuomotor goal-directed task which required participants to move their wrist to different target positions. The variable admittance scheme and both controllers were optimized in a group of ten healthy participants and then tested in six stroke patients.

## 2 Materials and methods

First, this section describes the implementation on the exoskeleton of the variable admittance scheme and the controllers. A short characterization of the variable admittance scheme is provided, followed by a description of how we evaluated the controllers in a behavioral task with healthy and stroke participants.

### 2.1 Apparatus

#### 2.1.1 The *eWrist*

**Figure 1:**
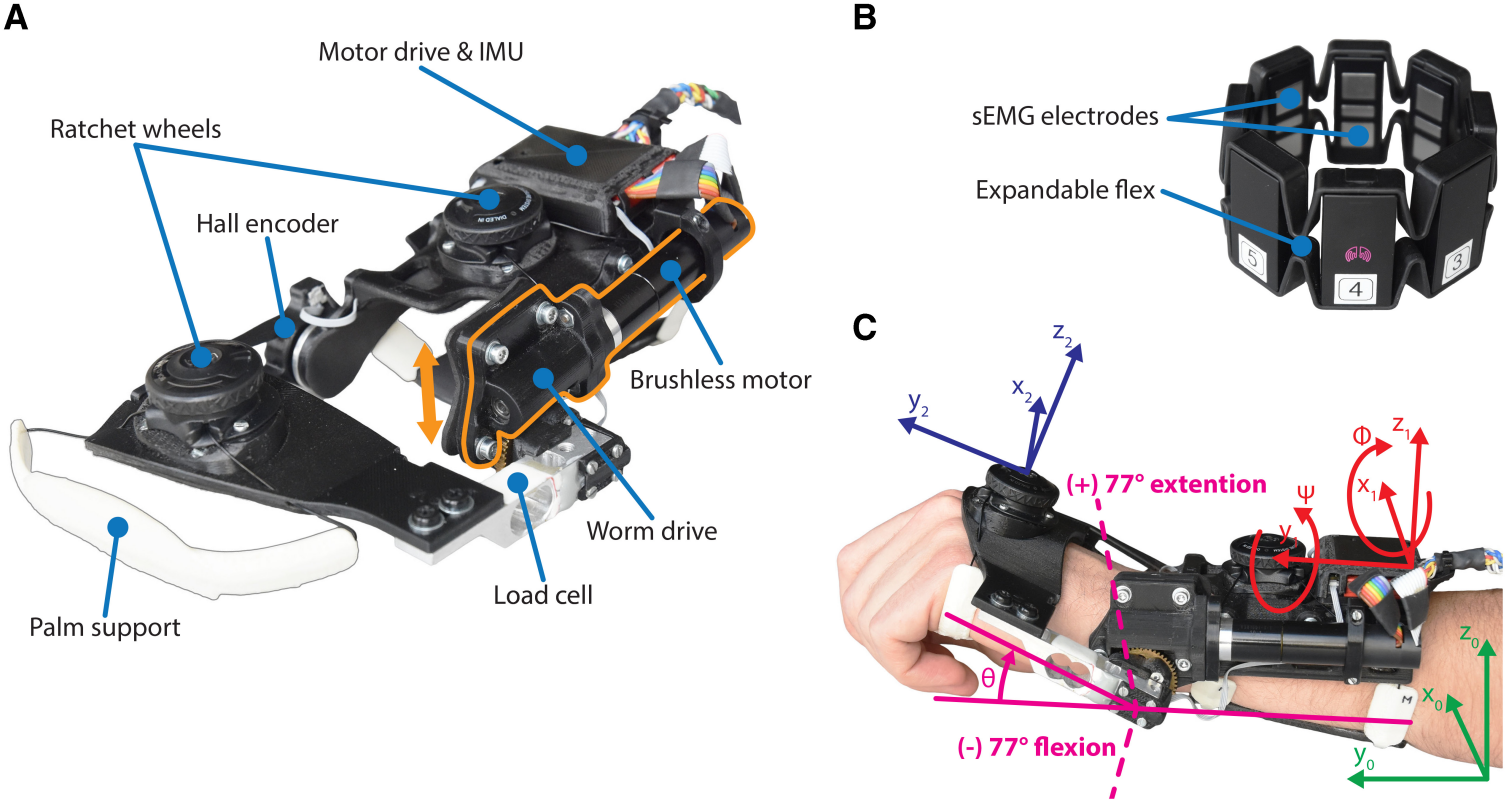
**(A)** The forearm module of the *eWrist*, where the motor and worm drive can be shifted up in order to uncouple the handle from the motor (see orange arrow). **(B)** The Myo gesture control armband from Thalmic Labs. **(C)** Illustration of the wrist angular position θ and the referentials used to compute *F*_*grav*_, i.e., the earth referential ℜ_0_ in green, the forearm module referential ℜ_1_ in red, and the hand referential ℜ_2_ in blue.

**Figure 2:**
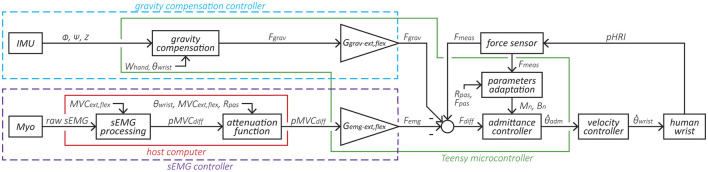
Block diagram of the variable admittance scheme and the two controllers. The elements within the purple dotted line form the sEMG-based controller, where the sEMG signal is normalized based on the participant's MVC and converted to a force *F*_*emg*_ via the gain *G*_*emg*_. The elements within the blue dotted line form the gravity compensation controller that produces a force *F*_*grav*_ based on the exoskeleton's orientation and normalized via the gain *G*_*grav*_. Subsequently, *F*_*emg*_ and *F*_*grav*_ are fed to the admittance controller. The elements within the green line are implemented in the Teensy microcontroller and run at 1 kHz, while the elements within the red line run on the host computer at 60 Hz.

The *eWrist* depicted in Figure 1A is a fully wearable 1 DOF force controlled wrist exoskeleton that actively supports extension and flexion movements (Lambelet et al., 2020, 2017). It actuates the wrist with a torque up to 3.7 Nm, an angular velocity up to 530°/s over a range of motion (ROM) of 215°. A load cell measures the force applied by the user on the handle. Absolute angular position and velocity are measured with a Hall encoder placed at the wrist axis and a Hall sensor integrated on the motor shaft, respectively. An inertial measurement unit (IMU) measures the orientation of the exoskeleton. Because of high reduction gear ratios, the transmission mechanism of the device is not backdrivable. The *eWrist* is fixed on the forearm and hand of the user as shown in Figure 1C. The variable admittance scheme is implemented in a real-time microcontroller (Teensy 3.2, MK20DX256 32 bit ARM Cortex-M4 72 MHz) and actuates the handle of the exoskeleton based on interaction forces as shown in Figure 2. During the visuomotor task the Teensy collects and transmits force, angular position/velocity and IMU data to a host computer via serial communication (USB).

#### 2.1.2 The Myo armband

The Myo armband was^1^ a commercially available device from Thalmic Labs. It measures sEMG activity on the forearm and consists of eight dry sEMG sensors. It has a sampling frequency of 200 Hz for raw sEMG data and communicates via Bluetooth Low Energy (BLE) making it easy to connect to other devices wirelessly. The raw data ranges from –128 to +128 and is unitless. The Myo armband can be easily donned, doffed, and adjusted to many forearm sizes (see Figure 1B).

### 2.2 Control

The core control of the *eWrist* is based on a variable admittance scheme that uses measured pHRI forces *F*_*meas*_ to generate a motion (see block *admittance controller* in Figure 2). The sEMG- and gravity-based controllers produce a mechanical support that is either based on the sEMG signal measured on the forearm with the Myo armband or based on the spatial orientation of the forearm module measured with an IMU. Both sEMG- and gravity-based controllers generate a fictive additional force (*F*_*emg*_ and *F*_*grav*_, respectively) that is subtracted from *F*_*meas*_, the result of which *F*_*diff*_ is the input to the variable admittance scheme, as shown in Figure 2. This additional force can be fine tuned with gains (*G*_*emg*_ and *G*_*grav*_). In this study, the sEMG- and gravity-based controllers were never used simultaneously along with the admittance controller, i.e., it was either the admittance + sEMG (cf. Equation 1) or admittance + gravity (cf. Equation 2).


(1) $$\begin{array}{l} F_{diff}=F_{meas}-F_{emg} \end{array}$$

or


(2) $$\begin{array}{l} F_{diff}=F_{meas}-F_{grav} \end{array}$$

Note that given our sign convention, *F*_*emg*_ and *F*_*grav*_ are being subtracted from *F*_*meas*_ to stabilize the support and prevent any runaway effects. For instance, in the case of the sEMG controller, a support is provided by the exoskeleton as long as the measured sEMG activity exceeds the measured interaction force.

#### 2.2.1 Variable admittance scheme

Admittance control receives a force input and outputs a motion in response as shown in Equation 3. Two parameters, namely virtual inertia *M* (*Nm*·*s*^2^/*rad*) and virtual damping *B* (*Nm*·*s*/*rad*) can be tuned to change the dynamic behavior of the exoskeleton. Equation 3 expresses the equation of motion in the time domain and its conversion to the Laplace domain with respect to angular velocity.


(3) $$M\ddot{\theta }+B\dot{\theta }=F\cdot L\;\overset{ℒ(\cdot )}{\Rightarrow }\;\omega =\frac{L}{Ms+B}\cdot F$$

where $\ddot{\theta }$ and $\dot{\theta }$ are the angular acceleration and angular velocity of the wrist in the time domain, respectively, ω the angular velocity in the Laplace domain, *L* the distance between the mechanical axis and the average pressure point of the hand on the handle (set at 8 cm), and *F* the force applied on the handle.

A discretized version of the admittance scheme is implemented in the Teensy microcontroller with the Tustin transformation (Lambelet et al., 2020). A variable admittance scheme is used to both reject disturbances in the pHRI, but also to render low inertia and transparent behavior of the device. To that end, *B* is dynamically adjusted either to dampen the system in case of instabilities or to free it during smooth motion (Dimeas and Aspragathos, 2016; Grafakos et al., 2016). The adaptation of *B* is represented by the block *parameters adaptation* in Figure 2 and is described in Equation 4.


(4) $$\begin{array}{l} B_{n}=B_{min}+G_{stif}\left(\theta \right)\cdot I_{s,n},\;n=\left\{1,2,3,...\right\}\\ M_{n}=M_{min} \end{array}$$

where *B*_*n*_ and *M*_*n*_ are the current damping and inertia values, respectively. *B*_*min*_ and *M*_*min*_ are the minimal damping and inertia values set at 0.04 (*Nm*·*s*/*rad*) and 0.004 (*Nm*·*s*^2^/*rad*), respectively, which render maximal transparency during smooth human-robot interactions. *G*_*stif*_(θ) is a non-linear gain depending on the angular position of the wrist θ, *I*_*s, n*_ the current index of stability, and *n* the control loop counter. In our implementation of the variable admittance scheme, *M*_*n*_ was kept constant and only *B*_*n*_ was dynamically adjusted (Dimeas and Aspragathos, 2016).

*G*_*stif*_(θ) expresses the change in passive stiffness of the wrist joint according to its angular position. The passive stiffness increases close to the limits of the joint's ROM (i.e., more likely to generate oscillations in the pHRI) and is the lowest around a straight wrist position. Therefore, by acting on *B*_*n*_, *G*_*stif*_(θ) dampens the system faster (i.e., to dissipate energy and attenuate oscillations) for large wrist angles than for small wrist angles. *G*_*stif*_(θ) changes continuously based on the wrist angle θ, is subject-dependent, and is set during a calibration phase. More information on *G*_*stif*_(θ) is provided in the Supplementary Equation S1. *I*_*s, n*_ is a recursive index which varies according to both the frequency and the magnitude of the oscillations measured in the force signal as shown in Equation 5.


(5) $$\begin{array}{l} I_{s,n}=I_{freq,n}\cdot I_{mag,n}+\lambda \cdot I_{s,n-1} \end{array}$$

where *I*_*freq, n*_ is the current frequency index, *I*_*mag, n*_ the current magnitude index, λ a parameter set at 0.7 that controls the frequency and magnitude parameters of the output *I*_*s, n*_, and *I*_*s, n*−1_ the previous index of stability.

*I*_*freq*_ encodes the frequency of oscillations, whereas *I*_*mag*_ encodes the magnitude of these oscillations as shown in Equations 6, 7. Both indexes are computed in real-time on the Teensy over a moving window of length *m* = 200 samples. The moving window moves in increments of eight samples since *I*_*s*_ is computed every eight loops of the admittance control loop (i.e., at 1,000/8 = 125 Hz) to limit impacting the assistance.


(6) $$\begin{array}{l} I_{freq,n}=\frac{\overset{m}{\underset{i=1}{\sum }}signChange_{i}}{\#signChange_{max}} \end{array}$$

where *signChange*_*i*_ is a boolean value and indicates whether the sign of the force changed at sample i in the moving window, and *#signChange*_*max*_ the maximum number of sign changes of the force in the moving window, which normalizes *I*_*freq, n*_ between 0 and 1, and which was determined empirically and normalized to m/8.


(7) $$\begin{array}{l} I_{mag,n}=\frac{\sqrt{\frac{1}{m}\left({\left(f_{h,n}\right)}^{2}+\cdots +{\left(f_{h,n-m}\right)}^{2}\right)}}{f_{max}} \end{array}$$

where *f*_*h, n*_ is the latest force sample, *f*_*h, n*−*m*_ the oldest force sample, and *f*_*max*_ the maximum value of the force which normalizes *I*_*mag, n*_ between 0 and 1, and which was determined empirically and set to 8 N.

**Figure 3:**
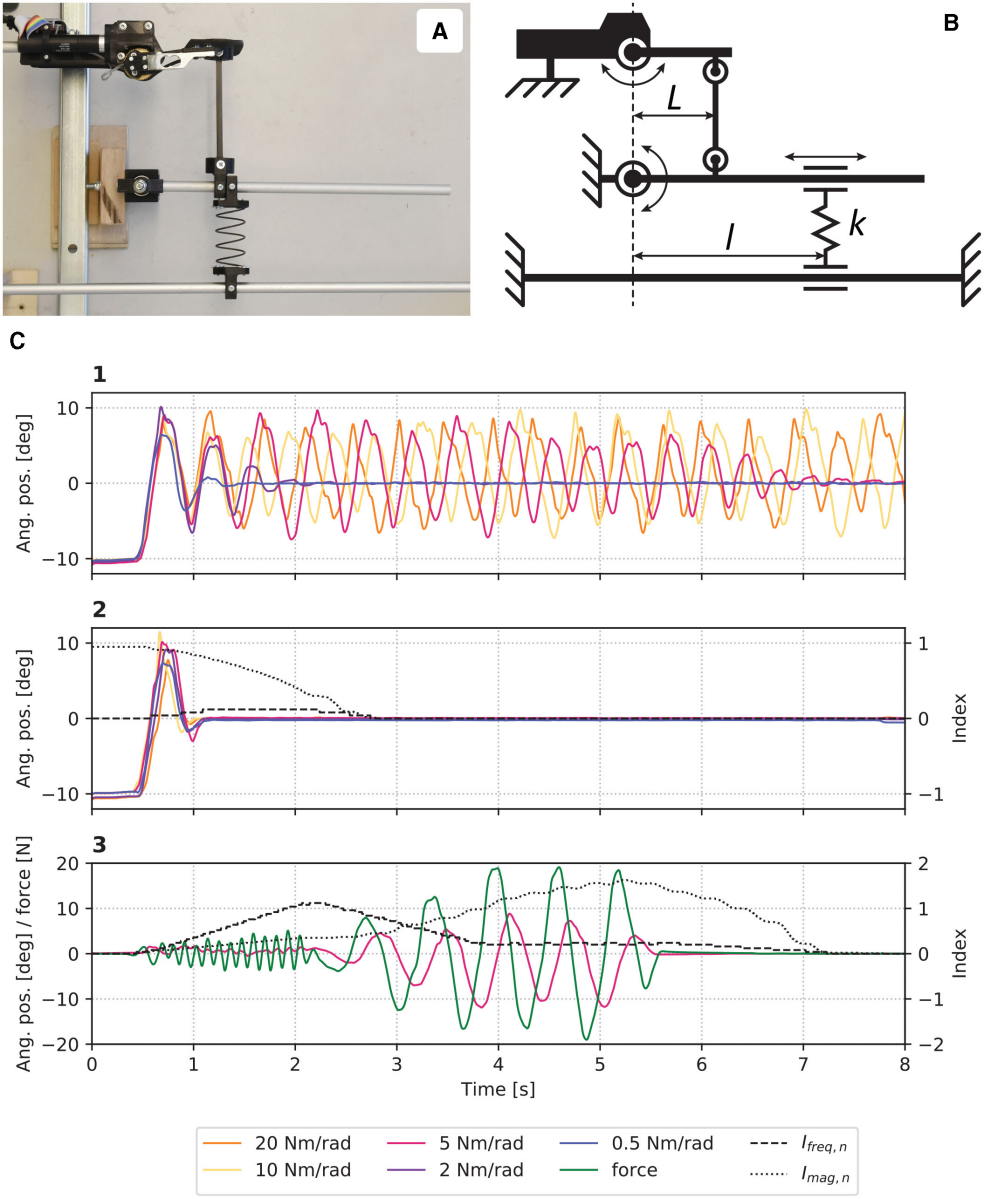
**(A)** Picture and **(B)** schematic of the experimental setup where stiffnesses perceived by the *eWrist* can be modulated by moving the spring *k* along *l*. **(C)** Results from the evaluation where in (1) *B*_*n*_ was kept constant, and in (2) *B*_*n*_ was adjusted dynamically. In (3), the system was manually excited with high frequency and magnitude oscillations.

The dynamic adaptation of *B*_*n*_ was evaluated in a setup where the *eWrist* was exposed to various stiffnesses. For this purpose, the forearm part of the device was firmly fixed and its handle linked to a rod. This rod was fixed to a rotating lever to which a spring of constant *k* = 698 N/m was attached as shown in Figures 3A, B. The spring could be moved along the lever in order to modulate the stiffness experienced by the exoskeleton from 0.5 to 20 Nm/rad. This range of stiffnesses is typical during active stiffening of the wrist joint (Halaki et al., 2006; Kuchenbecker et al., 2003; Leger and Milner, 2000). In Figure 3C, the handle was initialized to 10° in flexion and released to analyse the evolution of the movement when (1) *B*_*n*_ was kept constant at *B*_*min*_ and (2) *B*_*n*_ was adjusted dynamically as described in Equation 4. In (3), the handle was manually excited first with high frequency and low magnitude oscillations, and then low frequency and high magnitude oscillations.

In Figure 3C1, one observes that without a dynamic adaptation of *B*_*n*_, the device enters a resonant mode and cannot stabilize when the stiffness was set to 10 and 20 Nm/rad. However, the oscillations attenuate with lower stiffnesses. On the other hand, when *B*_*n*_ is dynamically adjusted, the system stabilizes for all stiffnesses as shown in Figure 3C2. Figure 3C3 shows how I_*freq, n*_ and I_*mag, n*_ respond to high frequency and high magnitude oscillations, respectively. Moreover, it takes about 2 s for both indexes to come back to their initial level once the excitation terminates.

#### 2.2.2 sEMG-based controller

The sEMG controller inputs an additional force *F*_*emg*_ to the variable admittance scheme as shown in Figure 2. First, raw sEMG data from the Myo armband are rectified and processed with a moving average filter (window length = 40 samples) followed by a Kalman filter^2^ to extract the envelope of the signal (cf. *sEMG processing* block in Figure 2). Kalman filtering on sEMG data offers low time lag and high computational efficiency as shown in Lyu et al. (2020). Two electrodes are assigned to measure the activity of extensor muscles, and another two electrodes assigned to measure the activity of flexor muscles. A weighted sum of the signals from each of these groups of two electrodes is performed and then normalized to the maximum voluntary contraction (MVC) (Corbett et al., 2011) separately for extension *MVC*_*ext*_ and flexion *MVC*_*flex*_. Then the difference between the normalized extension and flexion sEMG signal *pMVC*_*diff*_ is computed.

Secondly, *pMVC*_*diff*_ is attenuated by a non-linear gain *G*_*att*_(θ), which is a function of the wrist angle θ (cf. *attenuation function* block in Figure 2). Similar to *G*_*stif*_(θ), as the wrist angle reaches the limit of the joint's ROM, the passive stiffness increases and requires extensive muscle contractions to further move the wrist or simply hold the position. Therefore, the sEMG signal, and ultimately the supportive force *F*_*emg*_, needs to be attenuated for high wrist angles (i.e., higher than 80% of *ROM*_*pas*_). Consequently, *G*_*att*_(θ) boosts movement initiation for small wrist angles, but prevents excessive mechanical support close to the joint's limit. *G*_*att*_(θ) is subject-dependent and is determined during the calibration phase. It relies on *MVC*_*ext*_/*MVC*_*flex*_ and *ROM*_*pas*_, and two separate gains are used for extension and flexion [see Supplementary Equation S2 for more details on *G*_*att*_(θ)].

Finally, a constant gain *G*_*emg*_ (different for extension and flexion) transforms the unitless sEMG signal *pMVC*_*diff*_ into a force *F*_*emg*_. The decision to use one of the two gains is based on the sign of *pMVC*_*diff*_. A positive difference represents extension, whereas a negative difference represents flexion. *G*_*emg*_ was adjusted to provide appropriate mechanical support. The *sEMG processing* and *attenuation function* blocks within the red line in Figure 2 are processed in real-time at 60 Hz by the host computer. The time delay between the generation of raw sEMG and movement onset was evaluated over two separate measurements each consisting of 32 trials to 0.188 s, which is generally considered acceptable (Farrell and Weir, 2007). Note that the characteristics of the high frequency components of the sEMG signal is captured within the Myo armband, processed, averaged, and sent over BLE. Using averaged sEMG signals proved to work effectively with our simplistic approach that compares extensor and flexor muscle activity in the forearm.

#### 2.2.3 Gravity compensation controller

Similar to the sEMG controller, the gravity compensation controller inputs an additional force *F*_*grav*_ to the variable admittance scheme as shown in Figure 2. This controller continuously compensates the weight of the user's hand in extension or flexion based on the spatial orientation of the forearm module (ϕ, ψ, and *Z*) and the angular position of the wrist θ. Measurements from the IMU and the wrist angular encoder are used to compute *z*_*hand*_, the z-component of the normal vector to the hand *z*_2_ expressed in the Earth's referential ℜ_0_ (*x*_0_, *y*_0_, *z*_0_), as depicted in Figure 1C. First, spatial orientation of the hand referential ℜ_2_ relative to ℜ_0_ is calculated via the multiplication of *R*_*x*1_(ϕ), *R*_*y*1_(ψ), and *R*_*x*2_(θ). These three matrices encode for the rotation (relative to ℜ_0_) around the pitch (*x*_1_) and roll (*y*_1_) axes of the exoskeleton, and for the rotation (relative to the exoskeleton's referential ℜ_1_) around the wrist axis (*x*_2_), respectively. The result is then multiplied by *z*′ to extract the normal vector *z*_2_ as shown in Equation 8.


$$\begin{array}{l} R_{x1}\left(\phi \right)=\left[\begin{array}{ccc} 1 & 0 & 0\\ 0 & \cos\left(\phi \right) & -\sin\left(\phi \right)\\ 0 & \sin\left(\phi \right) & \cos\left(\phi \right) \end{array}\right]\\ R_{y1}\left(\psi \right)=\left[\begin{array}{ccc} \cos\left(\psi \right) & 0 & -\sin\left(\psi \right)\\ 0 & 1 & 0\\ \sin\left(\psi \right) & 0 & \cos\left(\psi \right) \end{array}\right]\\ R_{x2}\left(\theta \right)=\left[\begin{array}{c} 1\\ \sin\left(\theta \right)\\ \cos\left(\theta \right) \end{array}\right],\;z^{\prime }=\left[\begin{array}{c} 0\\ 0\\ 1 \end{array}\right] \end{array}$$

(8) $$\begin{array}{l} z_{2}=R_{x1}\left(\phi \right)\cdot R_{y1}\left(\psi \right)\cdot R_{x2}\left(\theta \right)\cdot z^{\prime }=\left[\begin{array}{c} -\sin\left(\psi \right)\\ -\sin\left(\phi \right)cos\left(\psi \right)\sin\left(\theta \right)\\ \cos\left(\phi \right)\cos\left(\psi \right)cos\left(\theta \right) \end{array}\right] \end{array}$$

*z*_*hand*_ is the z-component of *z*_2_ and varies between 0 and 1 according the hand's orientation as shown in Equation 9.


(9) $$z_{hand}={z^{\prime }}^{T}\cdot z_{2}=\cos(\phi )\cos(\psi )cos(\theta )$$

The gravity compensation force *F*_*grav*_ applied by the exoskeleton on the user's hand is computed as a fraction of the hand's weight *W*_*hand*_, as illustrated in Equation 10.


(10) $$\begin{array}{l} F_{grav}=z_{hand}\cdot W_{hand}=\cos\left(\phi \right)\cos\left(\psi \right)cos\left(\theta \right)\cdot W_{hand} \end{array}$$

Finally, a constant gain *G*_*grav*_ (different for extension and flexion) is used to fine-tune and adjust the mechanical support. When $\dot{\theta }$ is positive the extension gain is used, and when $\dot{\theta }$ is negative the flexion gain is used. The *gravity compensation* block within the green line in Figure 2 is processed in real-time at 1 kHz by the Teensy microcontroller.

### 2.3 Subjects

Ten healthy participants [seven males, mean age: 27.7 ± 3.8, ranging: (22, 33) years] and ten stroke survivors were recruited. In the healthy participant group, eight were identified as right-handed and two as ambidextrous according to the Edinburgh inventory (Oldfield, 1971). In the stroke survivor group, one withdrew for reasons unrelated to the study, one had very little sEMG activity, and two had very high co-contraction levels, which left six stroke survivors [four males, mean age: 57.3 ± 12.7, ranging: (40, 70) years] that performed the task. Details about stroke participants can be found in Table 1. The study was approved by the institutional ethics committee of ETH Zurich (2020-N-126). All subjects gave written informed consent in accordance with the Declaration of Helsinki before participating in the experiment.

**Table 1:** Details on stroke participants (*N* = 6).

**Subject**	**Age (years)**	**Sex**	**Time PS (month)**	**Stroke type**	**Imp. side**	**Handedness**	**FM-UE**	***ROM*_*act*_ (°)**	***ROM*_*pas*_ (°)**
S1	70	Male	167	Haem.	Left	Right	44	–33 / 42	–58 / 76
S2	54	Male	144	Haem.	Left	Right	43	–50 / 54	–68 / 74
S4	64	Male	162	Haem.	Right	Ambidextrous	52	–31 / 34	–71 / 60
S6	70	Male	52	Isch.	Left	Right	23	–45 / 11	–71 / 72
S7	40	Female	36	Haem.	Right	Left	22	–57 / 56	–77 / 78
S10	47	Female	59	Isch.	Right	Left	26	–52 / 22	–68 / 62

PS, poststroke; FM-UE, Fugl-Meyer for upper extremities; haem., haemorrhagic; isch., ischemic.

### 2.4 Experimental setup

**Figure 4:**
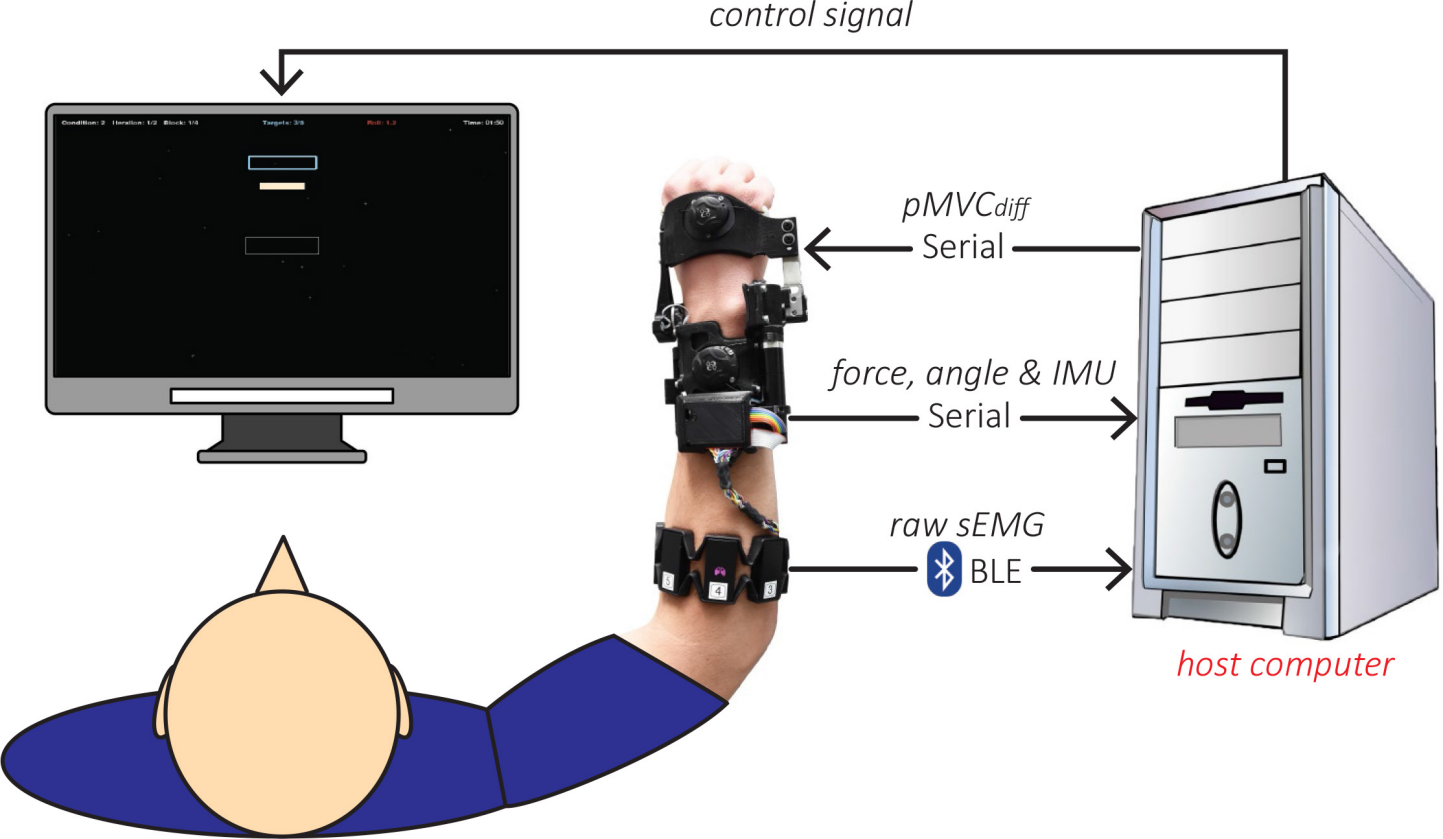
Experimental setup of the visuomotor task.

The experimental setup includes a host computer (see Figure 2) for data processing/recording and displaying the visuomotor task, the *eWrist* for actively supporting the wrist and measuring interaction force, angular position/velocity and IMU data, and the Myo armband for collecting sEMG signals on the forearm as depicted in Figure 4. Participants were seated on a chair in front of a screen with the *eWrist* and Myo armband mounted on their left forearm/hand for healthy participants and on their impaired forearm/hand for stroke participants. The forearm was placed on an armrest whose height was adjusted so that the shoulder was at 45° of abduction and the elbow formed an angle of 90°. Moreover, the hand protruded from the armrest so as to allow extension/flexion wrist movements.

### 2.5 Visuomotor task

**Figure 5:**
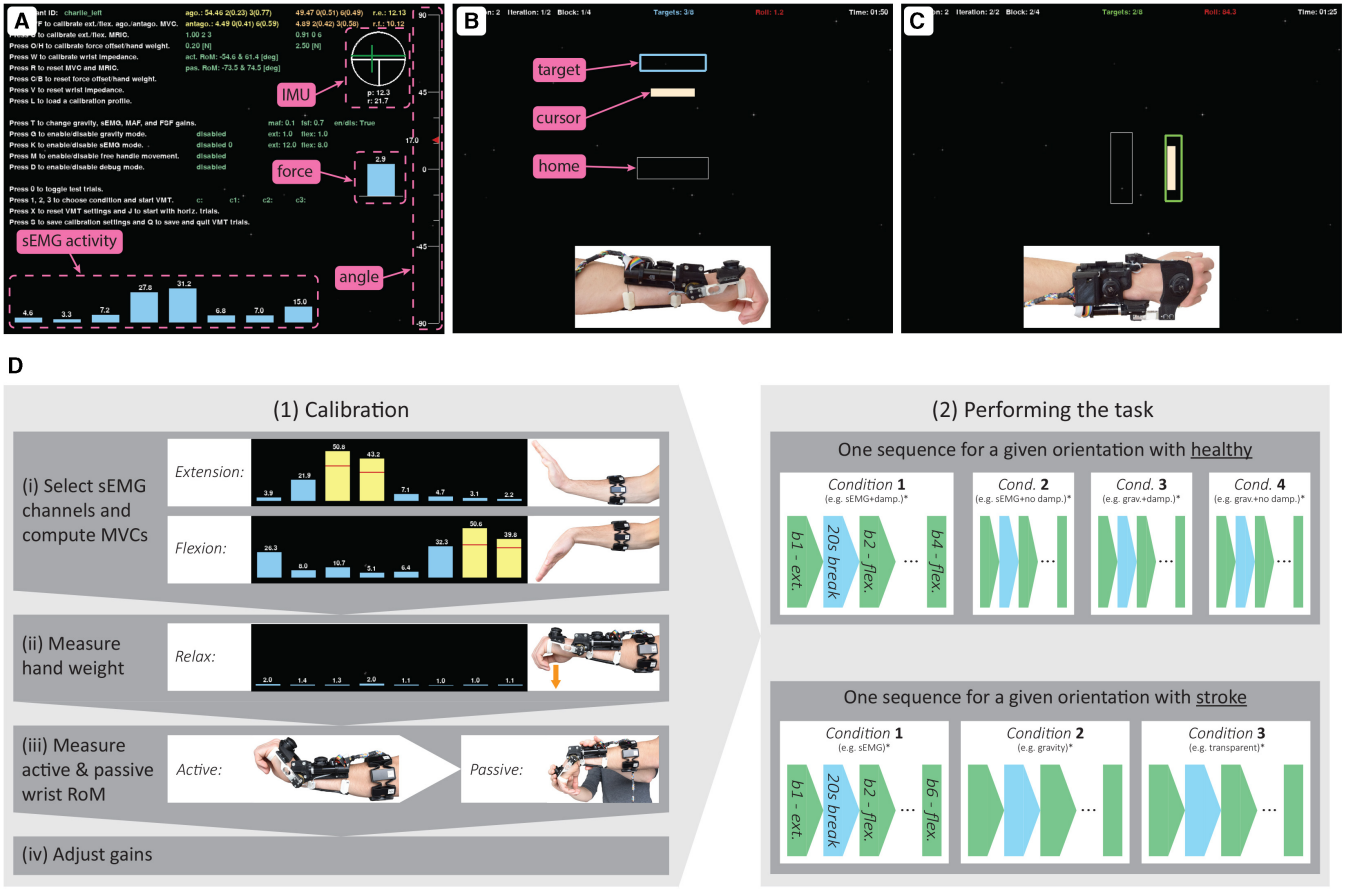
**(A)** Calibration window of the VMT where sEMG activity, force, wrist angle and IMU readouts are displayed in real-time. **(B)** VMT during vertical and **(C)** horizontal movements. **(D)** *Left:* calibration phase where in (i) MVC (indicated with a red bar) is measured on the two selected electrodes (in yellow) for extension and flexion separately, in (ii) low sEMG activity is required to assess *W*_*hand*_, in (iii) active and passive (with the help of the experimenter) ROM is assessed, and in (iv) the gains are adjusted. *Right:* description of the two different sequences for healthy and stroke participants. *The order of the conditions was pseudo-randomized across participants.

To assess the functionality of the variable admittance scheme and the controllers, a goal-directed experiment was developed in the form of a visuomotor task (VMT). The goal of the VMT was to reach targets with a cursor whose position is directly mapped in real-time to the angular position θ of the exoskeleton. The targets were placed at different positions on the screen (i.e., different θ) and required the participants to perform extension (θ > 0) and flexion (θ < 0) wrist movements to reach them (see Figure 1C). Once a target appeared, participants had 5 s to acquire it. When the cursor was in the target, the latter started to turn green as an indication of correct positioning. The target was acquired if the cursor remained 1 s within the target's boundaries. Once a target was acquired or 5 s elapsed, the target disappeared and the cursor was moved back into the home rectangle (see Figure 5B). The VMT was performed by both healthy and stroke participants, but the testing profile for each cohort was different. Before each testing session, a calibration phase was performed for each participant. The VMT was designed in Python 3.6 and implemented on the host computer running Ubuntu 18.04 LTS.^3^

#### 2.5.1 Calibration phase

The calibration phase depicted in Figure 5D consisted of (i) manually selecting two channels for extension and two channels for flexion, and determining the maximum voluntary contraction (MVC), (ii) assessing the weight of the user's hand, (iii) evaluating the active and passive ROM of the wrist joint, and (iv) adjusting the sEMG and gravity gains *G*_*emg*_ and *G*_*grav*_, respectively (see Figure 2).

##### 2.5.1.1 Selection of channels and MVCs

In order to obtain the largest sEMG difference *pMVC*_*diff*_ during extension (positive diff.) and flexion (negative diff.) movements, a compromise was made between selecting two channels with the highest sEMG activity for a given movement, but also the lowest agonist/antagonist overlap with the two other channels of the opposite movement. MVC was measured as the maximum sEMG activity that can be sustained for 1 s over an epoch of 5 s. MVC is computed for each selected channel individually and a weighted average is performed to obtain a single MVC value for extension *MVC*_*ext*_ and flexion *MVC*_*flex*_. Thereafter, sEMG signals are normalized to *MVC*_*ext*_ and *MVC*_*flex*_. During calibration, participants received real-time visual feedback of the activity of all 8 channels as shown in Figure 5A.

##### 2.5.1.2 Assessment of the hand's weight

The hand's weight *W*_*hand*_ was measured by the load cell when the participant was wearing the exoskeleton, the forearm placed horizontally and orientated for vertical hand movements (see Figure 5B), and θ set at 17° in extension (see Figure 1C). During the measurement, participants were asked to fully relax their wrist joint, which could be verified by checking sEMG activity.

##### 2.5.1.3 Evaluation of the wrist's ROM

First the active ROM *ROM*_*act*_ was assessed, and then the passive ROM *ROM*_*pas*_. The participant was wearing the device (which was uncoupled from the motor to allow a free wrist motion) and was asked first to fully flex and then fully extend the wrist. Maximal angles reached in extension and flexion were obtained from the real-time angle readout depicted in Figure 5A. For the passive ROM assessment, the wrist of the participant was manually flexed and extended by the experimenter until discomfort was reported.

##### 2.5.1.4 Adjustment of sEMG and gravity gains

*G*_*emg*_ and *G*_*grav*_ were fine-tuned to provide adequate mechanical support to the participant. While *G*_*emg*_ was adjusted to find a balance between increasing the normal excursion of the wrist and maintaining good control of the device, *G*_*grav*_ was set to accurately support the wrist weight in the horizontal position. Further fine-tuning was performed based on the participant's feedback.

#### 2.5.2 Assessment of variable admittance scheme with healthy participants

The functionality of the variable admittance scheme was evaluated with healthy participants performing the VMT. For this purpose, the task was performed with and without adaptation of the damping parameter *B*_*n*_ (damping factor). Both the sEMG and gravity controllers were evaluated (controller factor). In order to assess the influence of gravity, the task was executed in two different orientations, i.e., during vertical and horizontal wrist movements (see Figures 5B, C). The testing profile depicted in Figure 5D consisted of two sequences, one for each orientation. The first sequence was always performed in the vertical orientation followed by the horizontal orientation. A sequence was composed of four conditions in which the controller and damping factors were interchanged (i.e., sEMG+damping, sEMG+no damping, gravity+damping, and gravity+no damping) and the order of these factors was pseudo-randomized across participants. Each condition was composed of 4 blocks. A block included eight trials (i.e., eight targets to acquire), where the height of targets were set to 40%–80% of *ROM*_*pas*_ (i.e., four targets per height). The order of targets was pseudo-randomized. The first block of a condition was always extension trials followed by flexion trials, and continued in an alternating manner. Blocks were separated by a 20 s break.

#### 2.5.3 Assessment of controllers with stroke participants

The sEMG and gravity controllers were evaluated with stroke survivors performing the VMT. Both controllers were compared to a control condition later named “transparent mode”. In the transparent mode, the motor is physically uncoupled from the handle (see orange arrow in Figure 1A), which allows the latter to move freely in extension and flexion directions—the wrist joint is therefore not mechanically supported. The transparent mode is not a controller *per se*, but a control condition. Only the angular position of the wrist θ and IMU data are recorded. In this assessment, the testing profile was similar to the previous experiment (see Figure 5D) except for the following points: (1) the adaptation of *B*_*n*_ was always enabled, (2) a sequence consisted of three conditions, one for each control mode (i.e., sEMG, gravity, and transparent), and the order of the control modes was pseudo-randomized across participants, (3) a condition was composed of six blocks, and (4) a block consisted of eight trials with the height of targets set to 20%, 40%, 60%, and 80% of *ROM*_*pas*_ (i.e., two targets per height).

### 2.6 Evaluation metrics

During the VMT, all data generated by the *eWrist* and the Myo armband were continuously recorded and saved. To quantitatively assess the variable admittance scheme and the controllers, the following metrics were investigated:

- Maximal angular velocity ${\dot{\theta }}_{max}$
- Maximal angular acceleration ${\ddot{\theta }}_{max}$
- Normalized integrated interaction torque ${\hat{T}}_{int}$
- Normalized integrated jerk (NIJ)
- Number of acquired targets

#### 2.6.1 Maximal angular velocity and acceleration

${\dot{\theta }}_{max}$ and ${\ddot{\theta }}_{max}$ reflect the transparency of the device. The higher the velocity and acceleration, the more transparent the device. ${\dot{\theta }}_{max}$ and ${\ddot{\theta }}_{max}$ were calculated offline from θ via the FDM (finite difference method) described in Equation 11.


(11) $$\begin{array}{l} {\dot{\theta }}_{n}=\frac{\theta_{n}-\theta_{n-1}}{\Delta t},\;{\ddot{\theta }}_{n}=\frac{{\dot{\theta }}_{n}-{\dot{\theta }}_{n-1}}{\Delta t},\;n=\left\{1,2,3,...\right\} \end{array}$$

where θ_*n*_ / ${\dot{\theta }}_{n}$ and θ_*n*−1_ / ${\dot{\theta }}_{n-1}$ are the current and previous angular position / velocity measurements, respectively, *n* the control loop counter, and Δ*t* = *t*_*n*_−*t*_*n*−1_ the time difference between the current and previous timestamp. As the sampling time interval of the VMT, Δ*t* is not constant and varies around 0.017 s (60 Hz).

**Figure 6:**
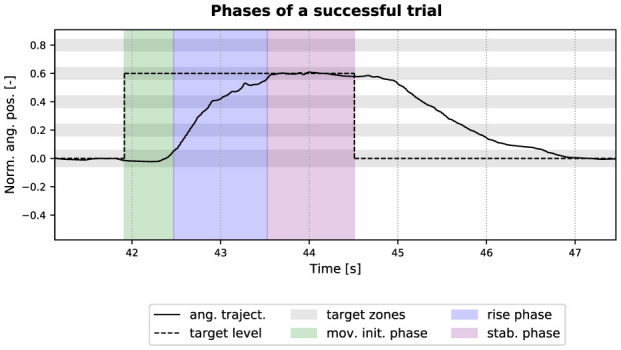
The three phases of a successful trial. The plain curve is the normalized (to *ROM*_*pas*_) angular trajectory $\hat{\theta }$, the dotted line is the target level, and the gray horizontal bars are the targets zones and the home zone. A successful trial (i.e., target acquired) is composed of the movement initiation phase (in green), the rise phase (in blue), and the stabilization phase (in purple). A target is reached when the cursor enters the corresponding gray zone.

Before the discrete differentiation to obtain ${\ddot{\theta }}_{n}$, ${\dot{\theta }}_{n}$ was low-pass filtered with a Butterworth filter.^4^ Both metrics were computed over the movement initiation and rise phases of the trial, and for trials where the target was reached (see Figure 6).

#### 2.6.2 Normalized integrated interaction torque

${\hat{T}}_{int}$ defined in Equation 12 is another indicator of the transparency of the device. The lower the interaction torque, the more transparent the device. ${\hat{T}}_{int}$ was computed over the movement initiation and rise phases of the trial, and for trials where the target was reached (see Figure 6).


(12) $$\begin{array}{l} {\hat{T}}_{int}=\frac{1}{\Delta t_{mir}}\cdot \overset{n_{end}}{\underset{i=n_{start}}{\sum }}T_{int,i}\Delta t \end{array}$$

where Δ*t*_*mir*_ = *t*_*n*_*end*__−*t*_*n*_*start*__ is the duration of the movement initiation and rise phases, *n*_*start*_ the starting sample point of the movement initiation phase, *n*_*end*_ the ending sample point of the rise phase, *T*_*int, i*_ the current interaction torque, and Δ*t* = *t*_*i*_−*t*_*i*−1_ the time difference between the current and previous timestamp.

#### 2.6.3 Normalized integrated jerk

The NIJ defined in Equation 13 is an empirical measurement of movement smoothness. The smaller the jerk, the smoother and less fragmented the movement (Hogan and Sternad, 2009). NIJ was computed over the whole trial, and for trials where the target was reached (see Figure 6).


(13) $$\begin{array}{l} NIJ=\sqrt{\frac{\Delta t_{trial}^{5}}{2\cdot s_{trial}^{2}}\cdot \overset{n_{end}}{\underset{i=n_{start}}{\sum }}jerk_{i}^{2}\Delta t} \end{array}$$

where Δ*t*_*trial*_ = *t*_*n*_*end*__−*t*_*n*_*start*__ and *s*_*trial*_ are the duration and path length of the trial, *n*_*start*_ and *n*_*end*_ the starting and ending sample points of the trial, *jerk* = *d*^3^θ/*dt*^3^ the 3rd derivative of the angular position θ, and Δ*t* = *t*_*i*_−*t*_*i*−1_ the time difference between the current and previous timestamp. $\frac{\Delta t_{trial}^{5}}{2\cdot s_{trial}^{2}}$ is a normalizing factor to obtain a unit-free measure (Teulings et al., 1997).

$\ddot{\theta }$ was low-pass filtered with a Butterworth filter (see text footnote 4) before the discrete differentiation to obtain *jerk*.

#### 2.6.4 Number of acquired targets

The overall performance of stroke participants in the task is assessed via the number of targets they could acquire.

### 2.7 Data analysis

A repeated measures ANOVA (α = 5%) was used to analyse data from healthy participants. The general model consisted in five factors with 2 levels each, namely: direction (extension/flexion), orientation (vertical/horizontal), controller (sEMG/gravity), height (40%/80%), and damping (with/without dynamic damping). Data were tested for normality. Moreover, the median across all trials of a given condition and subject was entered into the model. The median of these medians is then reported at the group level in the figures. The statistical analysis was performed on four sub-models, where a sub-model only considers a given direction and orientation. The rationale for this approach is that: (1) based on the orientation, gravity influences both controllers differently, (2) the different posture of the forearm in each orientation can influence the sEMG readout (due to different position of muscles relative to electrodes) and the ROM of the wrist joint, and (3) while the gravity controller supports the hand in the extension-vertical condition, it resists movement in the flexion-vertical condition. No statistical analysis was performed on stroke data because of the small sample size and high variability across participants, therefore, the results are descriptive.

### 2.8 Qualitative evaluation

Stroke participants completed two questionnaires during the testing session to quantify their subjective opinion of the different control modes and the visuomotor task in general. The first questionnaire is based on a 5 point Likert scale (Joshi et al., 2015) and assesses the mechanical support provided by both sEMG and gravity controllers. The questions were orientated around seven different aspects of the mechanical support, namely: force, speed, stability, consistency, lag, accuracy and ROM. Each of these aspects were evaluated independently. The second questionnaire is the Raw NASA-Task Load Index (RTLX) (Hart, 2006), which assesses the workload experienced during the VMT with the following aspects: mental/physical/temporal demands, performance, effort and frustration (Hart, 2006; Rubio et al., 2004). Each controller (sEMG and gravity) was assessed separately with both questionnaires, whereas the transparent mode was only evaluated with the RTLX. Finally, all participants could provide further comments at the end of the questionnaires.

## 3 Results

This section presents the behavioral results from healthy and stroke participants performing the VMT with the implemented controllers. The subjective evaluation of the controllers is also presented.

### 3.1 Assessment of variable admittance scheme with healthy participants

First, the results from the evaluation of the variable admittance scheme with healthy participants are presented. We predicted that adaptive damping would reduce jerk without generating higher interaction torques in the pHRI. We also predicted that adapted damping would not decrease maximal movement velocity and acceleration compared to the non-adaptive condition.

**Figure 7:**
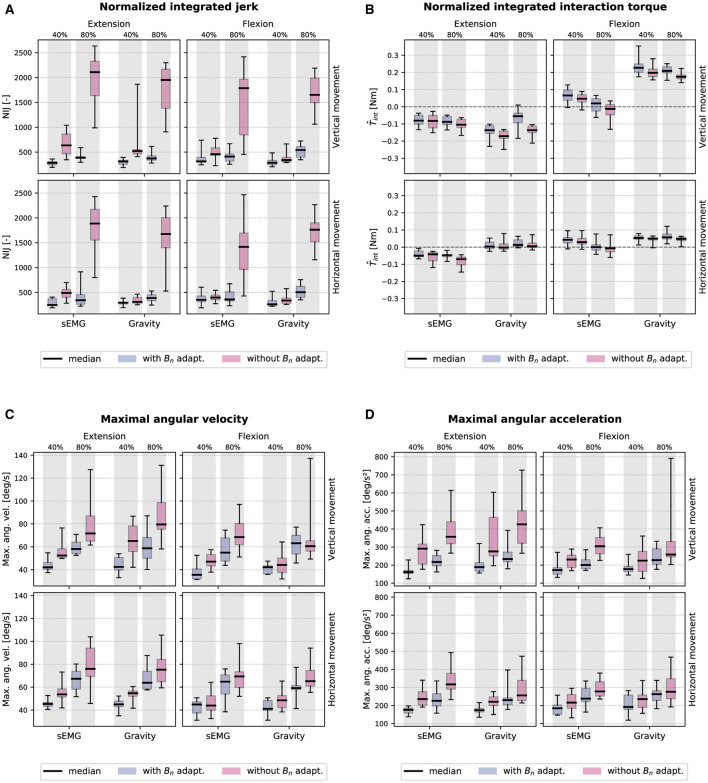
The boxplots show the median, interquartile range (IQR), and min./max. values of ten healthy participants for all factor permutations. The adaptive damping condition is shown in blue and the non-adaptive damping condition in pink. **(A)** Normalized integrated jerk (NIJ) and **(B)** interaction torque ${\hat{T}}_{int}$. **(C)** Maximal angular velocity ${\dot{\theta }}_{max}$ and **(D)** acceleration ${\ddot{\theta }}_{max}$.

Figure 7A presents the NIJ results at the group level. A significant main effect of damping (df = 1, *F* ≥ 50.569, *p* ≤ 0.001) and height (df = 1, *F* ≥ 71.632, *p* ≤ 0.001) factors is observed across all models. A strong interaction effect (df = 1, *F* ≥ 51.823, *p* ≤ 0.001) between damping and height was also observed across all models, which is clearly driven by increased jerk at the higher target level when *B*_*n*_ was not dynamically adapted. However, when *B*_*n*_ was actively adapted, jerk remained consistently low across all conditions. Moreover, while there was not a significant difference in jerk between the sEMG controller and the gravity controller for vertical orientations (df = 1, *F* ≤ 0.844, *p* ≥ 0.382), there was a significant difference for horizontal orientations (df = 1, *F* ≥ 5.115, *p* ≤ 0.050).

In Figure 7B, ${\hat{T}}_{int}$ varies substantially across conditions. Note that for a given direction, a negative interaction torque indicates that the device was supporting the movement, while a positive torque means that the hand was driving the movement. In the flexion-vertical-gravity condition, ${\hat{T}}_{int}$ is largely positive since participants had to counteract the upward supporting force imparted by the gravity controller. In all four models, ${\hat{T}}_{int}$ was significantly more positive in the adaptive damping condition (df = 1, *F* ≥ 5.298, *p* ≤ 0.047). This suggests that the user was slightly more supported (or less hindered) by the device when the damping was not dynamically adapted.

In Figure 7C, ${\dot{\theta }}_{max}$ was significantly greater at higher target levels compared to lower target levels in all models (df = 1, *F* ≥ 36.275, *p* ≤ 0.001), and was significantly greater when damping was not adaptive compared to adaptive damping (df = 1, *F* ≥ 6.455, *p* ≤ 0.039). The same pattern of results was observed for ${\ddot{\theta }}_{max}$ (see Figure 7D), main effect of target height: df = 1, *F* ≥ 10.160, *p* ≤ 0.015; main effect of damping condition: df = 1, *F* ≥ 8.391, *p* ≤ 0.023). Both the maximal angular velocity and acceleration results are inconsistent with our prediction that adaptive damping would not alter transparent rendering. In both metrics, the pHRI was consistently faster and more reactive when *B*_*n*_ was not dynamically adapted. Based on these results, we conclude that the implemented controllers offer a trade-off between faster but more jerky movements vs. slower but more stable movements. For stroke rehabilitation, stability is more important than speed, therefore, we favored the former for the assessment with stroke participants.

### 3.2 Assessment of controllers with stroke participants

**Figure 8:**
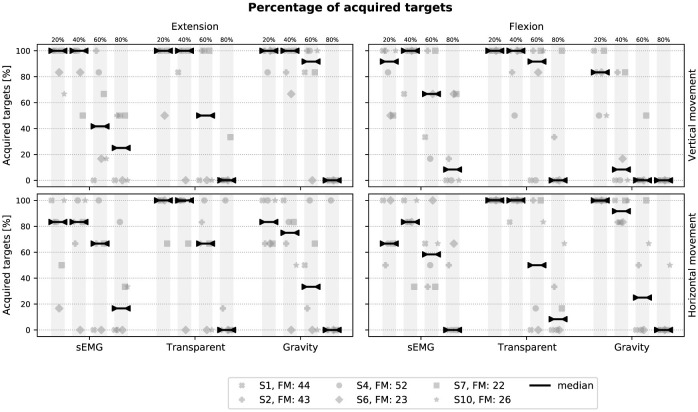
Percentage of acquired targets in stroke participants in all conditions. The median across participants is shown.

Figure 8 presents the percentage of acquired targets during the VMT for all stroke participants. Generally, the benefit of the device with the implemented controllers remains limited. In the transparent mode, participants had difficulties to reach the 80% targets especially in the extension-vertical condition since they had to move against gravity. This problem is somewhat less severe for the flexion-vertical condition and for both horizontal conditions. Whereas most participants benefited from the sEMG and anti-gravity support in the extension-vertical condition for the higher targets (especially S2, S4, and S7), less benefit was observed in other conditions. In particular, the anti-gravity controller negatively affected flexion movements since participants had to overcome the upward supporting force.

Although both controllers increased the ROM of participants and helped them to reach higher targets (see Supplementary Figure S1), they were still unable to stabilize their wrist to acquire the target within 5 s. This is particularly true for lower targets in the horizontal orientation with the sEMG-based controller. The loss of control could be the combined effect of not being influenced by gravity and the higher sensitivity of the controller at smaller angles due to *G*_*att*_(θ).

In the extension-horizontal-gravity condition, which requires only variable admittance control since no gravity compensation force was generated, participants also experienced difficulties to stabilize their wrist within the target boundaries. These instabilities could have been caused by an inability to relax the wrist joint, and could not be resolved by the dynamic damping. Moreover, as the horizontal condition was always performed after the vertical condition, fatigue and spasticity were more likely to be present during this phase, which could have resulted in increased stiffness of the joint. Nevertheless, participants S6 and S10 did appear to benefit from the mechanical support and acquired more targets compared to the transparent mode, especially in the extension-vertical condition (but also in both horizontal orientations). Interestingly, both S6 and S10 were amongst the most impaired of our participants, with FM-UE scores of 23 and 26 respectively.

#### 3.2.1 Subjective evaluation

**Figure 9:**
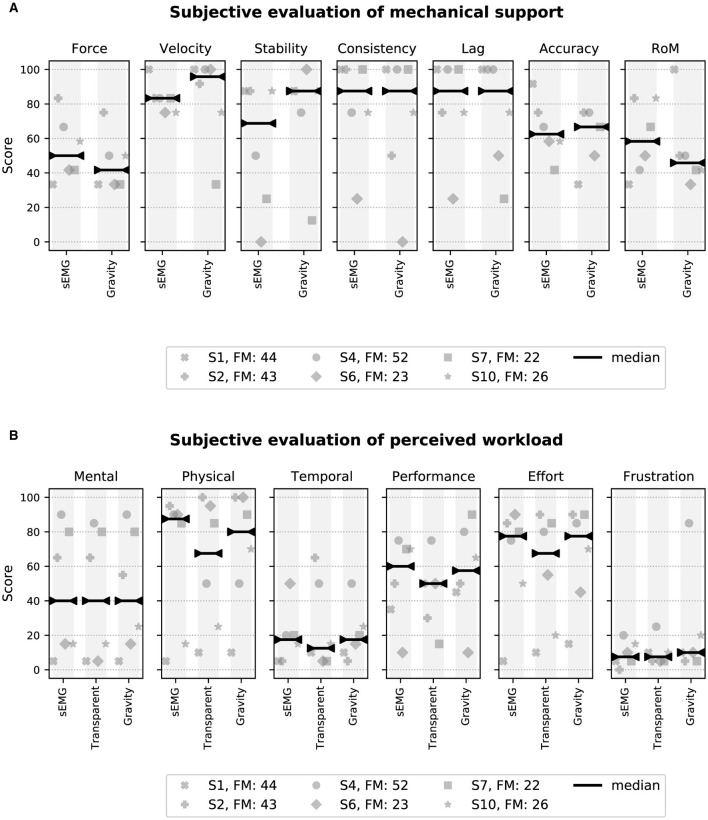
Score comparison derived from questionnaires for stroke participants where the median is shown. **(A)** Scores from the questionnaire assessing the mechanical support. The average score over all aspects is 66.1 ± 12.8 for sEMG and 64.1 ± 12.9 for Gravity. **(B)** Scores from the RTLX questionnaire. The average workload score excluding *Performance* (Grier, 2015) is 40.2 ± 25.2 for sEMG, 39.0 ± 21.4 for Gravity, and 44.8 ± 23.2 for Transparent.

A synthesis of the two questionnaires that stroke participants completed immediately after using a controller for the first time is presented in Figure 9.

Figure 9A shows the scores of the questionnaire assessing mechanical support from the *eWrist* during the VMT, which is based either on sEMG signals or on the orientation of the device. The score ranges from 0 to 100. A high score indicates that the aspect of the mechanical support being rated was appropriate. Based on the median score for each aspect, both the sEMG and gravity controllers were evaluated in a similar way. Generally, participants found that the supporting torque was too weak [*Force* median score (sEMG/gravity): 50.0/41.7], especially in the flexion-vertical-gravity condition where the device resisted the movement. However, they did rate the assistive movements as sufficiently fast (*Velocity* score: 83.3/95.8). Moreover, the sEMG controller was found to be less stable than the gravity controller (*Stability* score: 68.7/87.5). The support from both controllers was perceived to be consistent with the movement intention (*Consistency* score: 87.5/87.5), and the lag between the intention to move and the assistance provided by the device was sufficiently low (*Lag* score: 87.5/87.5). Because of a low supportive torque, participants tended to undershoot higher targets, and because of the high sensitivity of the controllers, they tended to overshoot lower targets, which impacted their accuracy (*Accuracy* score: 62.5/66.7). For the same reason, they found that the assistance was active across a range of motion that was too small (*RoM* score: 58.3/45.8).

Figure 9B shows the scores of the RTLX questionnaire, which also ranges from 0 to 100 and reflects the workload experienced during the task. The lower the score, the lower the perceived workload. A high score in *Performance* indicates that the participants felt that they were successful in performing the task. In most cases and based on the median score for each aspect, all three control modes were evaluated equally. *Mental, physical* and *effort* ratings (median scores for sEMG/Transparent/Gravity: 40/40/40, 87.5/67.5/80, and 77.5/67.5/77.5, respectively) exhibit large variability across participants. Generally, they found the task very demanding mentally and physically, most likely because of its difficulty (to reach 80% of the passive ROM) and its duration (~ 1.5 h). As a result of the perceived difficulty, they rated their performance to be rather low (*Performance* score: 60/50/57.5). All participants reported that they were not rushed (*Temporal* score: 17.5/12.5/17.5) during the task, nor frustrated (*Frustration* score: 7.5/7.5/10).

In the comments left by the participants, some reported that the scoring of *performance* and *effort* was mainly influenced by the difficulty of reaching higher targets. They also highlighted the resistance of the gravity controller in the vertical movement condition and stressed that it was difficult and unnatural to hold the forearm position for the horizontal movement condition. Apart from that, all participants expressed interest and motivation in the experiment, and reported that the task encouraged active participation and was challenging. None of them expressed any discomfort due to the device. Generally, participants considered that the duration of the testing session was adequate, however, S7 and S10 showed clear signs of fatigue at the end of the session. Finally, participants were asked to score their most favored (+1) and least favored (–1) control modes. As shown in Table 2, the sEMG controller was favored slightly more than the gravity controller or the transparent mode.

**Table 2:** Control mode preference of stroke participants.

**Subject**	**sEMG**	**Gravity**	**Transparent**
S1	+1	–1	0
S2	+1	0	–1
S4	0	+1	–1
S6	0	+1	–1
S7	0	–1	+1
S10	0	–1	+1
**Total**	**2**	**–1**	**–1**

## 4 Discussion

This study explored the implementation of a variable admittance scheme on a non-backdrivable portable wrist exoskeleton. In addition, an sEMG-based and a gravity-based controller were implemented in order to enhance the functionality of the wrist and promote voluntary effort. The variable admittance scheme and both controllers were first optimized with ten healthy participants performing a visuomotor task, and then evaluated in six chronic stroke patients performing the same task. The results with healthy participants showed that the variable admittance scheme could successfully and significantly improve the stability of the pHRI, but at the cost of a decrease in transparency. Furthermore, while both controllers improved the ROM of the wrist for stroke patients, the stabilization during target acquisition remained challenging. This was particularly true with the sEMG controller for the most distant targets, but also for near targets during horizontal wrist movements. Finally, the results also showed that patients with higher levels of impairment were more likely to benefit from the support provided by the *eWrist*.

### 4.1 Considerations on the variable admittance scheme

Humans are dynamic systems characterized by a time-varying impedance and their interaction with non-backdrivable haptic devices featuring admittance control can lead to instabilities. This usually occurs when the human limb stiffens to stabilize its motion (Ferraguti et al., 2019). In addition, during interaction with an unstable robot limb stiffness is increased by the central nervous system in order to reduce these external perturbations (Burdet et al., 2001). As the stiffness of the user is not directly measurable, the controller cannot easily account for this issue.

In this work, the frequency and magnitude of the interaction force signal was analyzed to detect instabilities and dampen the system accordingly using a variable admittance scheme. The implemented variable admittance control scheme acted solely on the damping parameter to attenuate instabilities. Damping is a velocity-dependent parameter that dissipates energy in the pHRI. Therefore, increased damping leads to more energy dissipation and the restoration of stable behavior, but at the same time imposes increased resistive force during steady velocity movements. Previous studies have shown that only adapting the damping and not the inertia parameter could unbalance the admittance dynamics and affect the usability of the robot (Lecours et al., 2012). It was suggested that adapting the inertia term (instead of damping) is more beneficial for low-effort movements, and only affects acceleration/deceleration phases (Dimeas and Aspragathos, 2016). However, the optimal strategy depends on robot structural dynamics, the limitations of the actuators and sensors, and the implementation of the admittance control scheme, which highlights the need to make design decisions on a case-by-case basis (Topini et al., 2022).

We based our decision on initial experiments where we investigated the effect of adapting: (1) only the damping, (2) only the inertia, and (3) both terms while keeping a constant ratio between them. These experiments revealed that adjusting only the damping was most promising because it had the highest impact on stability compared to the two other options. Nevertheless, deeper consideration of the other options, especially with regard to parameter tuning, might have also revealed positive effects on both stability and transparency.

Several alternative strategies could have been considered. First, the position of the robot can be used to dissociate low frequency components of the human movement from high frequency components caused by the instability (Ryu et al., 2008). However, the admittance control scheme described in Equation 3 acts as a low pass filter for high frequency position oscillations. Consequently, the reduced magnitude in the frequency domain deteriorates the detection of instability, which is de facto not a suitable approach for admittance control (Dimeas and Aspragathos, 2016). Moreover, whereas previous studies have used a fast Fourier transform (FFT) to analyse the frequency domain (Dimeas and Aspragathos, 2016; Ryu et al., 2008), the present work implemented a less computationally demanding algorithm for use on a microcontroller. The algorithm simply counts the number of times the force signal changes sign in a moving window of *m* = 200 samples, while this window moves in increments of eight samples. The window length m and the increment size were optimized to obtain sensitive and reactive damping adaptation, but also to not significantly slow down the control loop running at 1 kHz. The execution time to compute the index of stability *I*_*s*_ is around 200 μs. These parameters were tuned on a single healthy subject and never changed for the behavioral assessments. Although user-dependent adjustment of these parameters would improve performance, the algorithm appeared to be a reliable and suitable solution for embedded systems with limited computing power as shown in Figure 3C. Note that the gains *G*_*stif*_, *G*_*att*_, *G*_*emg*_, and *G*_*grav*_ are subject-dependent and were always calibrated before each test session for healthy and stroke participants.

Second, the implemented method does not prevent instability but only eliminates the negative effects of oscillatory behavior. Therefore, instead of acting upon the instability retroactively, other methods analyzed co-activation level in the muscles in order to detect an increase of stiffness in the limb and proactively dampen the system (i.e., before the disturbance occurs) (Grafakos et al., 2016; Castellini et al., 2014; Gallagher et al., 2014; Raiano et al., 2020). By comparing the co-activation level in the forearm (extensor vs flexor) between the movement initiation/rise phases and the stabilization phase of a trial (see Figure 6), a higher co-activation level was observed in the latter phase (see Supplementary Figure S2). This demonstrates an increase of stiffness in the wrist joint during target acquisition. Such a strategy proved to be robust with healthy subjects manipulating end-effectors. It would be interesting to test a similar strategy with stroke survivors who exhibit irregular sEMG patterns.

Third, our feed-forward approach of adapting the variable gain *G*_*stif*_(θ), which is subject-dependent and dampens the system more rapidly for higher wrist angles, was motivated by the increase in stiffness of the wrist joint close to the limit of its ROM. This assumption proved to be correct as shown in Figure 7A where the pHRI was significantly more jerky for the most distant targets. This gain accounts for the passive stiffness of the joint related to its biomechanical properties, however, stroke survivors may exhibit an involuntary increase in stiffness over the whole ROM because of tremor or spasticity that may evolve over the course of rehabilitation. In such cases, it might be promising to explore whether a feedback approach analyzing co-activation level or abnormal contractions in the muscles could improve stability of the control mechanism.

In summary, our variable admittance control strategy, which can be implemented on a microcontroller, reduced involuntary oscillations caused by changes in wrist stiffness. This was achieved at the cost of reduced transparency, which was still sufficient to allow functional movements. Nevertheless, future work might make use of muscular co-activation detected from the sEMG signals to dampen the system either proactively or via a co-activation-specific feedback mechanism.

### 4.2 Considerations on the sEMG controller

The performance of the sEMG-based controller with stroke participants was surprisingly lower for near targets in horizontal orientations compared to the other control modes as shown in Figure 8. This decrease in performance results from a loss of control due to higher sensitivity of the controller (see Supplementary Figure S3) where jerk is more important for the sEMG controller compared to the gravity controller in these conditions). The higher sensitivity of the sEMG controller for near targets comes from the combined effect of *G*_*stif*_(θ) and *G*_*att*_(θ), which confer less damping and a more reactive response to the sEMG signal for small angles. The gain *G*_*att*_(θ) models the increase of sEMG production required to move the wrist close to the limits of its ROM, and prevent excessive mechanical support that would push the wrist beyond these limits. Although *G*_*att*_(θ) is subject-dependent and based on the calibrated MVC and passive ROM, its combined effect with *G*_*emg*_ can result in an overly sensitive controller at small wrist angles. Moreover, since *G*_*att*_(θ) is based on isometric contractions, it can introduce a systematic error when applied under dynamic conditions (Clancy and Hogan, 1997). With *G*_*emg*_ a trade-off had to be found in order to increase the ROM while maintaining good controllability.

The sEMG-torque relationship is complex and is influenced by many factors such as electrode placement relative to the innervation zone, muscle length, cross talk from nearby muscles, and number of motor units recruited (Farina et al., 2001). Moreover, several of these factors vary non-linearly with respect to movement velocity and joint position (Farina, 2006; Solomonow et al., 1991). The simple and straightforward approach adopted in this work was motivated by (1) a belief that the human central nervous system could compensate for a less accurate torque estimate provided by the robot as long as the latter is physiologically coherent, and (2) an envisioned implementation on an embedded system with limited processing power (Lenzi et al., 2012). For optimal performance of the sEMG-based controller, the electrodes must be carefully selected in order to maximize *pMVC*_*diff*_ during extension and flexion movements. Two electrodes were chosen for each direction so as to limit the effect of single electrode variations and thus to capture a more general pattern of activation. However, this strategy reaches its limits in the case of systematic co-contractions in the forearm, and consequently the activation patterns between extension and flexion movements cannot be sufficiently dissociated. In this study, the electrodes were selected by the experimenter, however, an automatic selection that minimizes overlap between extension and flexion activations could be implemented.

Finally, one assumption was that the sEMG-based controller might further excite the system in case of instabilities in the pHRI. As the user fights against the oscillations, they produce counterproductive sEMG patterns that are picked up by the controller and further excites the system. However, our results show that jerk is not significantly different between the sEMG and the gravity controller (see Figure 7A) suggesting that this is unlikely to have occurred. An explanation could be that given our setup and filtering process, the readout of sEMG signals was too slow to pick up fast oscillating patterns in the muscle activity, and thus could not influence the controller. This problem could arise with faster sampling rates, however, there are studies suggesting that sEMG-based admittance controllers with high sampling rates (e.g., 1 kHz) enhance stability, as they can detect the user's intention before human active force is measured, thereby minimizing response delays and reducing oscillatory behavior by synchronizing the human-robot interaction (Xie et al., 2021).

### 4.3 Considerations on the behavioral evaluations

In this work, the implemented controllers were evaluated during both vertical and horizontal wrist movements in order to assess the influence of gravity on the control of a portable exoskeleton. With healthy participants, this influence can be observed in the absolute value of the interaction torque ${\hat{T}}_{int}$, which was generally lower for horizontal movements as for vertical (see Figure 7B). This discrepancy is normal for the gravity controller but requires further explanations for the sEMG controller. In the extension-vertical condition, the hand had to be moved against gravity, which triggered more muscle activation and thus generated more support from the device (i.e., negative ${\hat{T}}_{int}$). This support is less pronounced in the extension-horizontal condition since less muscle activation was required to move the hand. A similar general trend is observed with ${\dot{\theta }}_{max}$ and ${\ddot{\theta }}_{max}$ (see Figures 7C, D). Especially in the extension-vertical conditions, the higher muscle activation and the anti-gravity supportive torque moved and accelerated the user's hand faster. Moreover, as the MVC calibration was solely performed in the vertical orientation, the performance of the sEMG controller could have been affected when performing the task in the horizontal orientation. Indeed, the position of muscles relative to the electrodes might have shifted due to the supination of the forearm (Kim et al., 2018). Finally, as the gravity controller depends on θ, this resulted in less support for larger angles as shown in Figure 7B for the extension-vertical condition.

The fatigue of stroke patients significantly affected the sEMG controller (Farina et al., 2001). Especially for the most distant targets, the fatigue added to the difficulty of the task resulted in more pronounced levels of co-contraction that ultimately decreased the efficiency of the controller (see Supplementary Figure S2). Moreover, actively holding the forearm for the horizontal condition added an extra contribution to fatigue. To counterbalance the effect of fatigue, the order of controllers in the task was changed across participants. The width of the target, which is a critical factor in determining task difficulty, was empirically set with healthy participants performing the task. It reflects an unimpaired ability level that stroke survivors should have when using the device. Moreover, the difficulty to reach the most distant targets was largely expressed in the workload feedback with the *Physical* and *Effort* aspects (see Figure 9B). In these two aspects, the transparent mode scores better (i.e., lower workload) than both controllers. This could explain a lower efficiency of the controllers for distant targets or an unsatisfied expectation toward the controllers. Finally, the large variability in some of the aspects of the workload scores may reflect a different level of involvement of participants in the task.

### 4.4 Limitations and future directions

There were a couple of limitations in our subjective evaluation of the controllers. Given our testing profile, the questionnaires for a given controller were always completed by the participant after the first sequence of vertical trials. This permits (1) to assess the condition in which the controllers support the wrist the most (i.e., during vertical movements), and (2) to obtain the most accurate and vivid feedback. However, the first control mode to be assessed does not benefit from a prior comparison, and the horizontal orientation is not represented in the subjective feedback. Moreover, the assessment of the mechanical support could have been laborious for participants with somatosensory deficits. Unfortunately, this aspect of their impairment was unknown. Half of the stroke survivors tested in this study presented moderate to low deficits in wrist function, and therefore felt more impaired by the device than helped. We should have not only recruited more impaired patients in the acute/sub-acute phase, but also a larger sample size. The small sample size and the wide impairment range of the tested cohort explains the low statistical power and large variability in the results. Testing a larger sample size would have allowed us to determine from which level of deficit a patient could benefit most from the device. Furthermore, the stroke survivors recruited in this study were volunteers and highly motivated, so may not be representative of the broader stroke population.

Future work could focus on combining features from both controllers in order to enhance the mechanical support. For instance, the intention of the patient could be captured through the sEMG signal to enable/disable the support of the gravity controller. This could apply when the sEMG signal is too weak and noisy for proportional control, but the intention could still be picked up via classification or regression methods (Khokhar et al., 2010; Ameri et al., 2019). In the same vein, the spatial orientation of the device could be used to adapt the gains of the controllers (especially sEMG) to make the pHRI more stable. As envisioned in this study, the developed controllers and algorithms should remain simple and efficient enough to be implemented in embedded systems. In this regard, the host computer in this study (cf. outlined in red in Figure 2) has already been implemented in the microcomputer of the *eWrist* (Lambelet et al., 2020). Moreover, our controller has the potential to extend to hand and grasp functions using end-effector robots, where assist-as-needed therapy can benefit from sEMG feedback and enhanced stability (Xie et al., 2021; Topini et al., 2022).

## 5 Conclusion

In the context of non-backdrivable exoskeletons for rehabilitation therapy, it is important that the physical human-robot interaction remains reactive and stable. However, instabilities can occur when the limb of the user stiffens to stabilize its motion. In this paper, we implemented a variable admittance control scheme together with an sEMG-based controller that promotes voluntary effort, and with a gravity compensation controller that supports weakness in the wrist joint. We have demonstrated that the implemented control scheme remains stable during a passive stiffening of the wrist joint, but impacted the transparency of the device. Moreover, we have shown that our controllers could enhance the capability of stroke survivors in the most extreme wrist positions even though stabilizing the device within a given target remained challenging. This may have been perceived as requiring a high physical effort, but this is not necessarily a disadvantage, as the purpose of the device is to facilitate the patient's voluntary effort to perform movements that are not possible without the support. Finally, this work has drawn attention to the influence of gravity on the proportional control of a portable exoskeleton, paving the way for further development in that field.

## Data Availability

The raw data cannot be made publicly available since the participants did not consent to sharing or reuse. Further enquiries should be directed to the corresponding author.

## Footnotes

^1^The Myo armband was discontinued in October 2018.

^2^*KalmanFilter* from the Python library *pykalman* was used.

^3^Processor: Intel(R) Core(TM) i7-4710MQ CPU @ 2.50GHz, RAM: 16GB.

^4^2^*nd*^ order 2 Hz cut-off frequency.
