# Supplementary material for: Variable admittance control with sEMG-based support for wearable wrist exoskeleton
Source: Front Neurorobot. 2025 Sep 1;19:1562675. doi: 10.3389/fnbot.2025.1562675 (PMC12434121; doi:10.3389/fnbot.2025.1562675)
Supplement: Supplementary file 1 [file Supplementary_file_1.zip › supplementary_data_sheet/supplementary_files/frontiers2024_supplementary_material_v1.pdf]

Supplementary Material

1 SUPPLEMENTARY EQUATIONS

1.1 Details on $G_{stif}(\theta)$

$G_{stif}(\theta)$ takes into account the passive ROM of the wrist ROM_{pas} and the passive force F_{pas} applied by the hand on the handle measured at four different angular positions, namely at 20% and 80% of ROM_{pas} for extension and flexion separately. As described in Equation S1, $G_{stif}(\theta)$ acts linearly on the damping of the system for angles smaller than 80% of ROM_{pas}, but quadratically for larger angles.

$$G_{stif}(\theta) = \begin{cases} \left|a_1\theta^2 + b_1\theta + c_1\right|, & \text{if } \theta < 0.8\theta_{mpf} \\ |a_2\theta + b_2|, & \text{if } 0.8\theta_{mpf} \leq \theta \leq 0.8\theta_{mpe} \\ \left|a_3\theta^2 + b_3\theta + c_3\right|. & \text{if } \theta > 0.8\theta_{mpe} \end{cases} \tag{S1}$$

where a_1, b_1, c_1, a_2, b_2, a_3, b_3 and c_3 are based on ROM_{pas} and F_{pas}, and are determined during the calibration phase. θ_{mpf} and θ_{mpe} are the maximal passive angles reached in flexion and extension, respectively, such that $\left|\theta_{mpf}\right| + |\theta_{mpe}| = ROM_{pas}$. According to our angle convention (see Figure 1C), θ_{mpf} is negative and θ_{mpe} positive, however, for the computation of $G_{stif}(\theta)$ and in order to deal only with positive angles, an offset of 77°was added to θ, θ_{mpf}, and θ_{mpe}.

1.2 Details on $G_{att}(\theta)$

$G_{att}(\theta)$ aims to linearize the non-linear relationship between the generation of forearm sEMG signals and the angular position of the wrist joint. $G_{att}(\theta)$ takes into account the passive ROM of the wrist ROM_{pas} and the MVC in extension MVC_{ext} and flexion MVC_{flex}. Two separate gains are used for extension and flexion, i.e. when θ is positive (extension) or negative (flexion) (see Figure 1C). As described in Equation S2, the inverse of linear or quadratic functions is taken based on the absolute value of θ to attenuate the sEMG signal.

$$G_{att}(\theta) = \begin{cases} (a_1\theta^2 + b_1|\theta| + c_1)^{-1}, & \text{if } |\theta| < 0.2|\theta_{mp}| \\ (a_2|\theta| + b_2)^{-1}, & \text{if } 0.2|\theta_{mp}| \leq |\theta| \leq 0.8|\theta_{mp}| \\ (a_3\theta^2 + b_3|\theta| + c_3)^{-1}. & \text{if } |\theta| > 0.8|\theta_{mp}| \end{cases} \tag{S2}$$

where a_1, b_1, c_1, a_2, b_2, a_3, b_3, and c_3 are based on ROM_{pas}, MVC_{ext} and MVC_{flex}, and are determined during the calibration phase. θ_{mp} is the maximal passive angle reached in flexion or extension.

1.3 Details on the co-activation level

The co-activation level (CL) defined in Equation S3 was computed over the movement initiation/rise phases CL_{mir} and the stabilization phase CL_{stab} (see Figure 6), and only for trials where the target was acquired. Thereafter, the ratio $\frac{CL_{stab}}{CL_{mir}}$ was analysed.

$$CL = \frac{1}{\Delta t_{ph}} \cdot \sum_{i=n_{start}}^{n_{end}} A_{e-f} \Delta t \tag{S3}$$

where $\Delta t_{ph} = t_{n_{end}} - t_{n_{start}}$ is the duration of the movement initiation/rise phases or the duration of the stabilization phase, n_{start} the starting sample point of the phase, n_{end} the ending sample point of the phase, A_{e-f} the overlapping activity between $pMVC_{ext}$ and $pMVC_{flex}$ (i.e. the smaller of the two at a given sample point), and $\Delta t = t_i - t_{i-1}$ the time difference between the current and previous timestamp. CL can vary from 0 (no overlapping) to 1 (full overlapping).

2 SUPPLEMENTARY RESULTS

The following figures present additional results on the assessment of the controllers performed with stroke participants. The figures always show the median and individual data of the six stroke survivors for all factor permutations (i.e. extension/flexion directions, vertical/horizontal orientations, sEMG/gravity controllers and no support, and 20%/40%/60%/80% target heights).

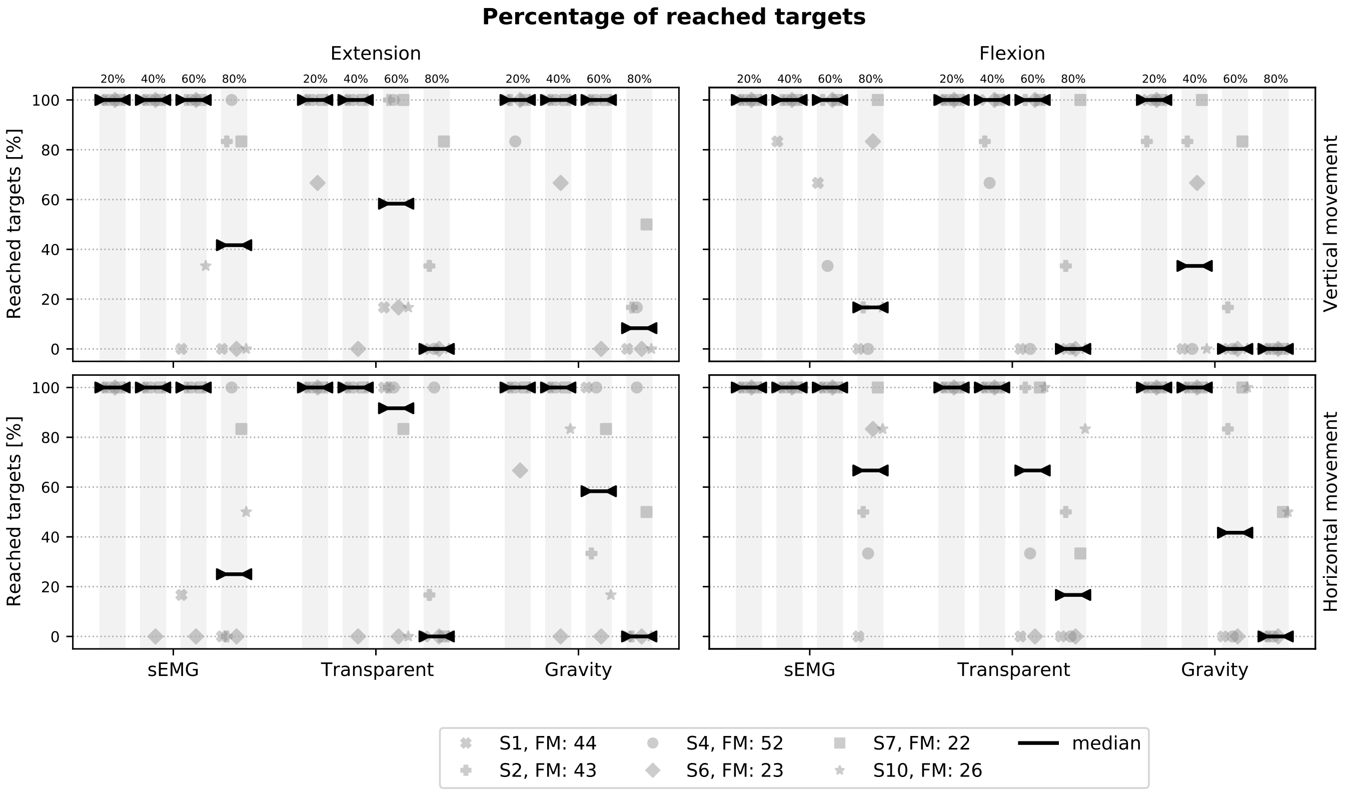

Figure S1. Percentage of reached targets in stroke participants in all conditions. The median across participants is shown.

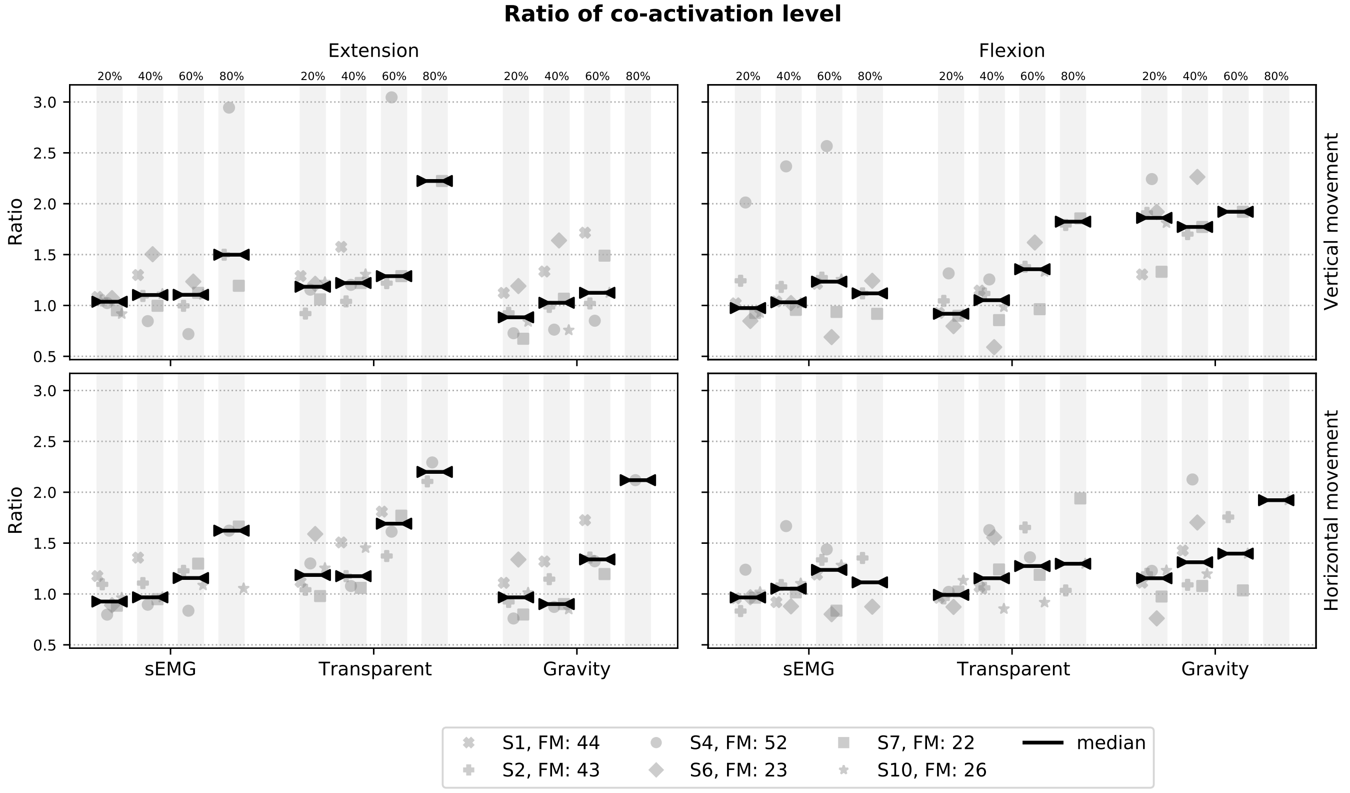

Figure S2. Ratio of co-activation level between the stabilization phase and the movement initiation/rise phases of a trial. The median across participants is shown. The co-activation level is computed as described in Eq. S3 and was evaluated only for trials where the target was acquired.

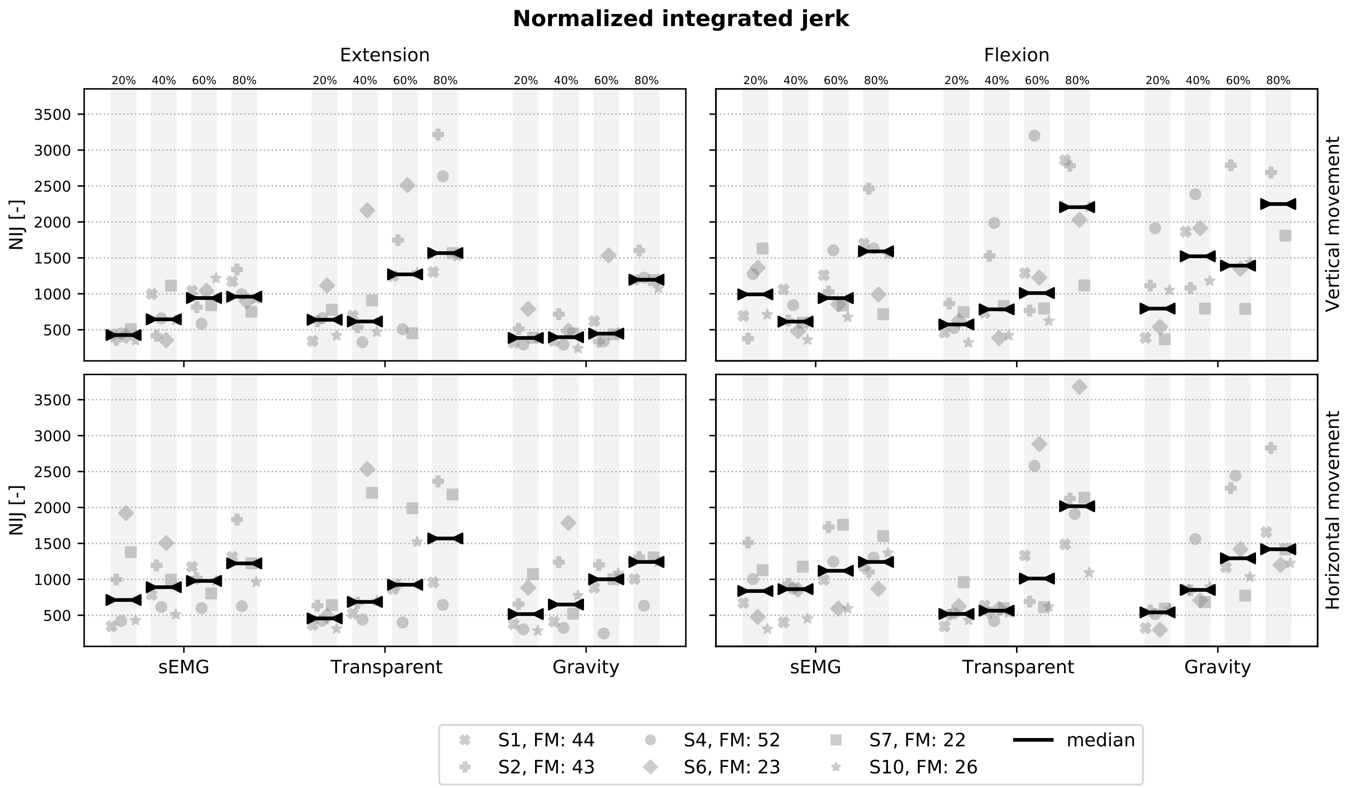

Figure S3. Normalized integrated jerk (NIJ) in stroke participants in all conditions. The median across participants is shown. The NIJ is computed as described in Equation 11 and was evaluated only for trials where 60% of the target's height was reached (in order to have more data).
