# Supplementary figures and images for: Variable admittance control with sEMG-based support for wearable wrist exoskeleton
Source: Front Neurorobot. 2025 Sep 1;19:1562675. doi: 10.3389/fnbot.2025.1562675 (PMC12434121; doi:10.3389/fnbot.2025.1562675)

### Normalized integrated jerk

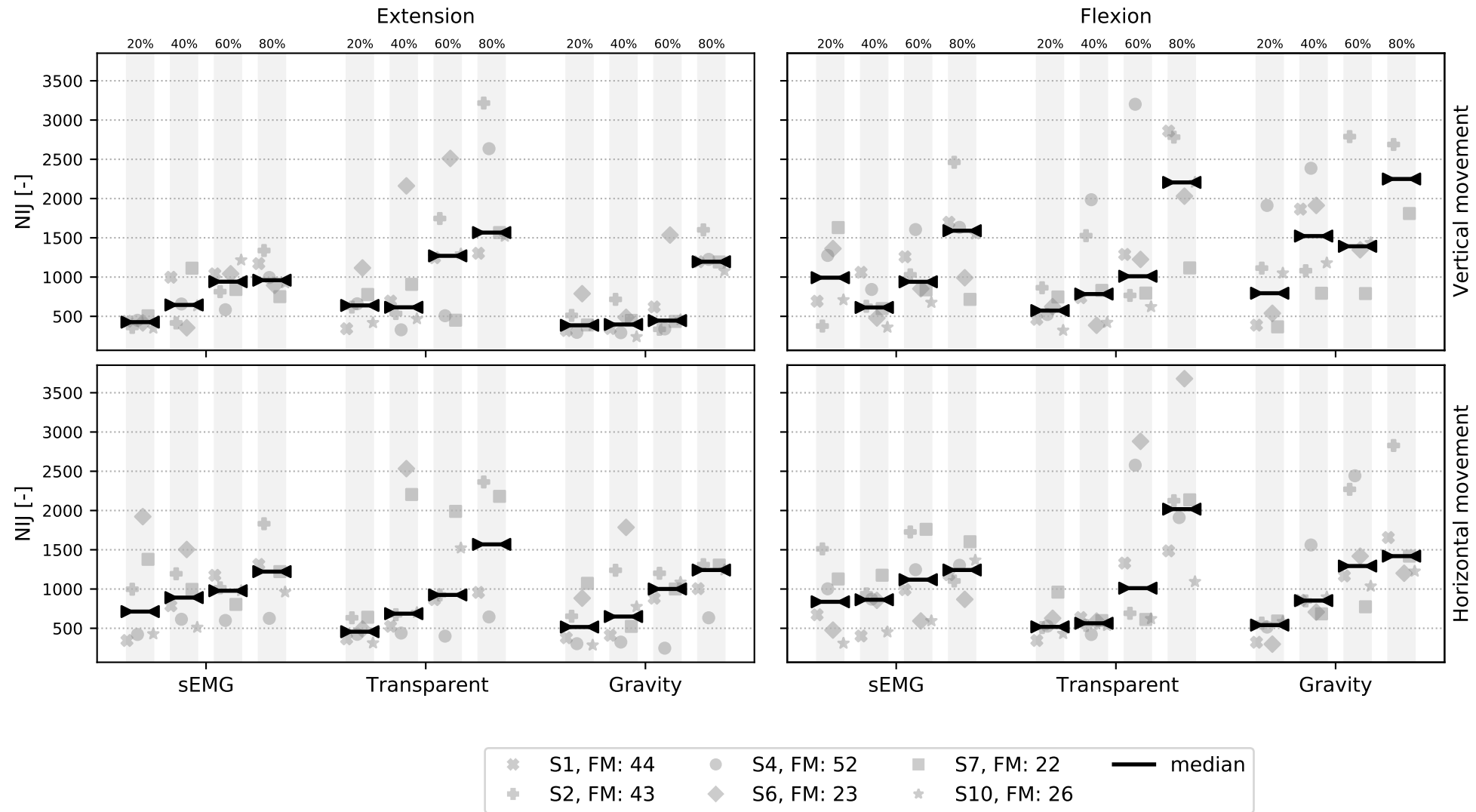

Supplement: Supplementary file 1 [file Supplementary_file_1.zip › supplementary_data_sheet/supplementary_files/ni_jerk_stroke.pdf]

# Percentage of reached targets

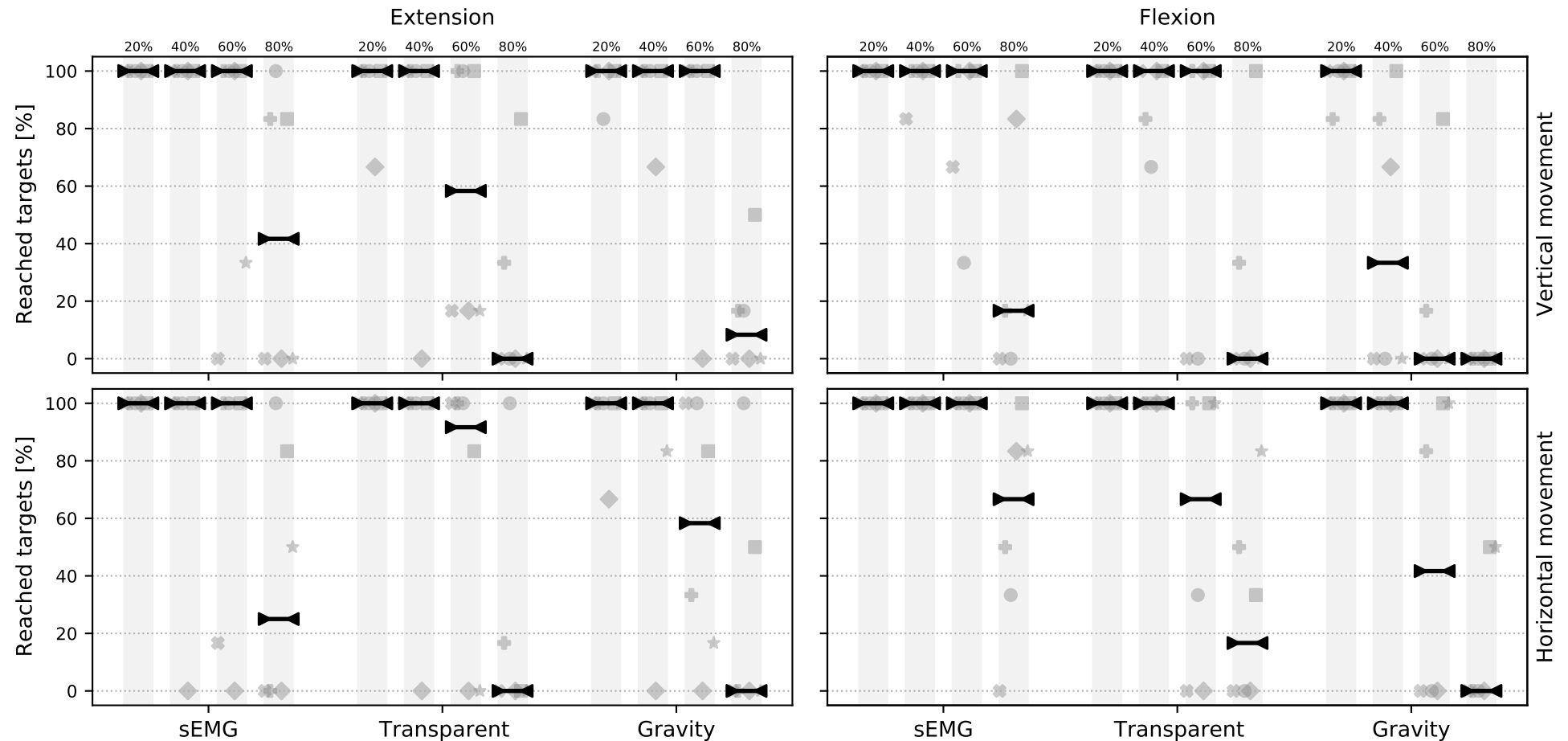

Supplement: Supplementary file 1 [file Supplementary_file_1.zip › supplementary_data_sheet/supplementary_files/perc_reached_stroke_bottom.pdf]

## Ratio of co-activation level

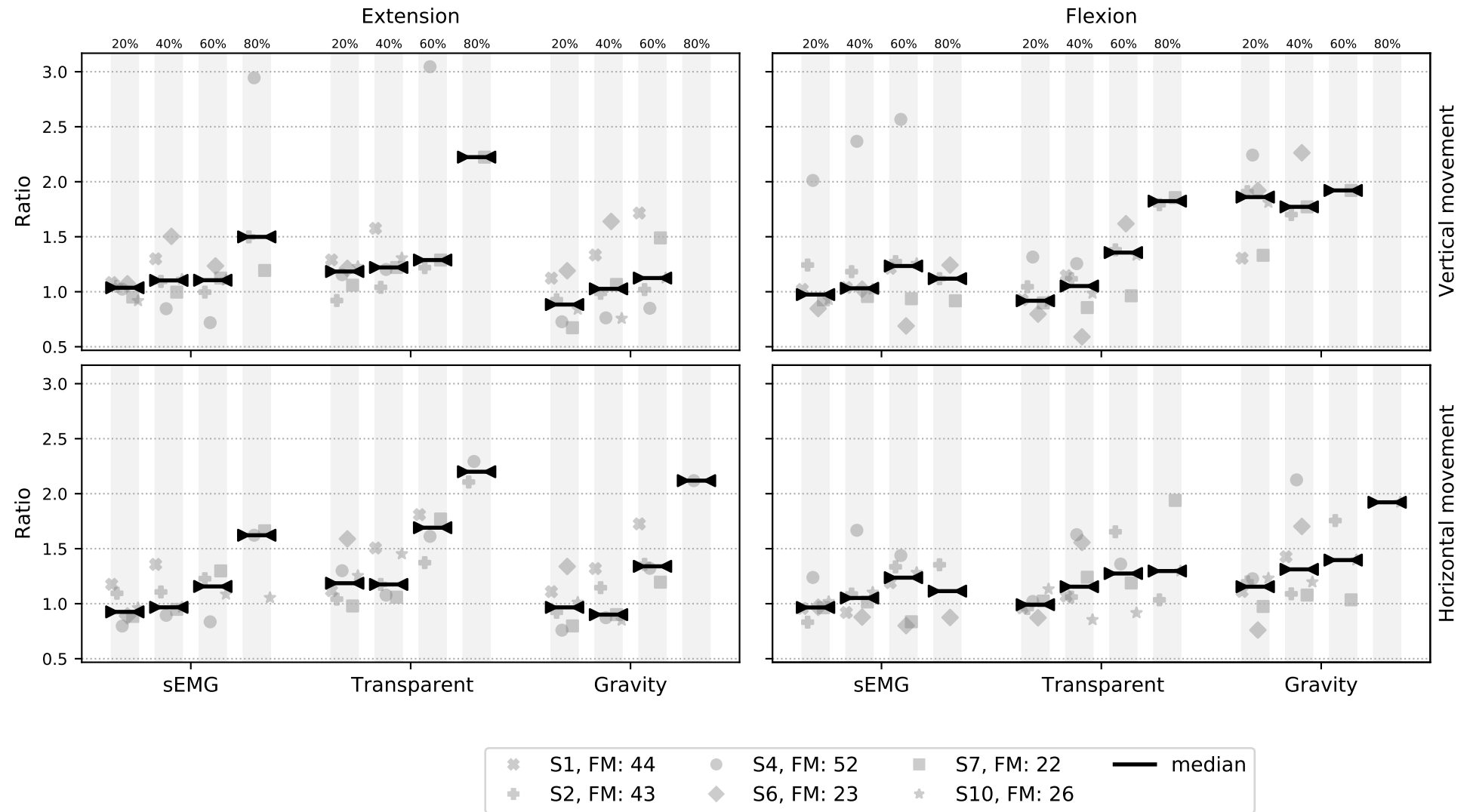

Supplement: Supplementary file 1 [file Supplementary_file_1.zip › supplementary_data_sheet/supplementary_files/ratio_coact_level_stroke.pdf]
